# Assessment of two minimally invasive methodologies for sex identification in the European eel, *Anguilla anguilla*

**DOI:** 10.1111/jfb.70361

**Published:** 2026-02-12

**Authors:** Michael J. Williamson, Jack A. Brand, Kevin Hopkins, Luke O'Connor, Matthew W. Perkins, Christopher Sergeant, Simon Spiro, Taina Strike, Jessica Whinfield, Rosie S. Williams, Ethan Wrigglesworth, Rosalind M. Wright, Adam T. Piper

**Affiliations:** ^1^ Institute of Zoology Zoological Society of London London UK; ^2^ Department of Genetics, Evolution and Environment University College London London UK; ^3^ Environment and Sustainability Institute University of Exeter Cornwall UK; ^4^ Department of Wildlife, Fish, and Environmental Studies Swedish University of Agricultural Sciences Umeå Sweden; ^5^ Zoological Society of London London UK; ^6^ The Harry Butler Institute Murdoch University Murdoch Western Australia Australia; ^7^ Taronga Conservation Society Australia Mosman New South Wales Australia; ^8^ Environment Agency Feering UK; ^9^ Scottish Centre for Ecology and the Natural Environment, School of Biodiversity, One Health and Veterinary Medicine University of Glasgow Glasgow UK

**Keywords:** anguillid eel, fish, molecular markers, non‐sacrificial, sex determination, ultrasound

## Abstract

Sex is an important driver of variation in behaviour, ecology and physiology. Sex identification in the Critically Endangered European eel (*Anguilla anguilla*) currently requires fish sacrifice, or the use of morphological differences such as body length, which can be inaccurate in certain habitats and at intermediate body lengths. Non‐lethal tools for accurate sex identification in this species are therefore required. Here, we test the efficacy of two minimally invasive methods of sex identification: (1) ultrasonography and (2) molecular markers taken from pectoral fin clips. Both methodologies were applied to riverine European silver eel and compared to histological sex identification. Ultrasonography accurately identified the gonads in female eel, with males identified by the absence of detectable gonadal structures. The level of expression of three molecular markers (*dcn, LOC111853410*, *kera*) previously used to identify sex in Japanese eel (*Anguilla japonica*) were evaluated. There was no significant differential expression of the three molecular markers between sexes, suggesting that these markers cannot be accurately used to identify sex in European eel. Our findings suggest that minimally invasive imaging using ultrasonography can be a reliable tool for identifying sex in early‐stage adult migratory European eels, with particular use at intermediate sizes (400–500 mm) and animals in habitats where migration potential is limited and may regularly exceed typical growth sizes. This technique can be highly valuable for studies that address ecological, behavioural, conservation and management issues in this Critically Endangered species.

## 1 INTRODUCTION

Life‐history processes and resource needs often vary between the sexes, resulting in different patterns of behaviour, habitat use, movement dynamics, distribution and abundance (Giroux et al., 2021; Korstian et al., 2013; Schuett et al., 2010; Smith et al., 2019; Tarka et al., 2018; Wearmouth & Sims, 2008). In fishes, sex‐specific differences in physiology and behaviour to anthropogenic disturbance and may be particularly pronounced. For example, pollutants often accrue at different levels between the sexes (Burger et al., 2007; Komarnicki, 2000; Madenjian et al., 2016), which can lead to sex‐specific neurotoxic effects (Gade et al., 2021). Males and females may have variable physiological responses to pollutants, particularly androgenic pollutants (Bertram et al., 2015; Milla et al., 2011; Saaristo et al., 2013), which may lead to differential impacts on metabolism, behaviour, growth, reproduction and survival (Adeogun et al., 2020; Erikstad et al., 2013). Other anthropogenic pressures, such as fishing and climate change, often differentially impact males and females with the potential to alter sex ratios within fish populations (Bade et al., 2019; Edmands, 2021; Geffroy & Wedekind, 2020; Ogburn, 2019; Ospina‐Álvarez & Piferrer, 2008; Uusi‐Heikkilä, 2020) which may have significant negative impacts on sexual selection processes, population growth and recruitment (Uusi‐Heikkilä, 2020). As such, accurate sex identification is a critical part of fish management and conservation (Holleley et al., 2023; Literman et al., 2014) as well as vital for basic understanding of ecology, evolution and behaviour (Conde et al., 2010; Gantchoff et al., 2019; Hess et al., 2018; Maiorano et al., 2017).

Among fish species, sex can be determined by both environmental and genetic factors (Horiuchi et al., 2022; Kitano et al., 2024; Nakamura, 2010). In the European eel (*Anguilla anguilla*), sex is environmentally, rather than genetically, determined (Geffroy & Bardonnet, 2016). Although the exact mechanisms of sex determination are not fully understood, it is thought that growth rate and density are significant drivers (Crowley et al., 2022; Davey & Jellyman, 2005; Geffroy & Bardonnet, 2016). Genetic testing for sex chromosomes is a common method for identifying sex in wildlife (Alacs et al., 2010; Palmer et al., 2019). However, species which have environmental sex determination, such as the European eel, often lack sex chromosomes, and therefore sex cannot be identified using this method (Shen & Wang, 2018).

In some fish species, sex can be identified using unambiguous secondary sexual characteristics, such as size or colouration patterns (Holleley et al., 2023; Scarsella et al., 2018), even in those species with environmental sex determination. However, silver European eels do not exhibit obvious secondary sexual characteristics and it is difficult to accurately distinguish males from females on the basis of their morphology (du Bureau Colombier et al., 2015; Tesch, 2003). Morphological differences in body length can be used to tentatively identify sex in European eels, with individuals of a total length above 500 mm designated as females, and those below 460 mm designated as males. However, extreme values outside of these ranges occur in both males and females (Boulenger et al., 2015; Denis et al., 2022), with the range from 400 to 500 mm considered unpredictable for sex determination (du Bureau Colombier et al., 2015; Tesch, 2003). Further, in systems with restricted or no seaward migration routes, such as lakes or reservoirs, individuals can reach larger sizes, which exceed the proposed length brackets for sex determination, and may have exceptionally fast growth rates due to optimum habitat and food resources (Piper et al., 2020; Simon, 2007; Trancart et al., 2018). For example, a recent study tagged 185 European eels in a landlocked reservoir with animals having a mean total length of 960 mm (standard deviation [SD] ± 68) with a minimum size of 820 mm (A.T. Piper, personal communication). In addition, a previous study by Piper et al. (2020) found lengths of adult eels at two landlocked drinking water reservoirs in the UK ranged from 832 to 1032 mm and from 732 to 1047 mm. Accordingly, atypical growth rates and maximum size make sex identification using morphological differences in body length unreliable in such waterbodies. There may be additional morphological differences between sexes in European eels, such as eye size and pectoral fin shape, but these are also impacted by genetics, age and the environment, and therefore cannot be used to reliably differentiate the two sexes (Hsu et al., 2023; Tesch, 2003). Currently, the primary method for sex identification is gonad assessment, which requires fish sacrifice (du Bureau Colombier et al., 2015; Höhne et al., 2023) or invasive sampling of the gonads endoscopically (da Silva et al., 2018; Kucharczyk et al., 2016; Macrì et al., 2014). Given that European eels are considered Critically Endangered by the International Union for the Conservation of Nature (IUCN) (Pike et al., 2020), that sex identification is essential for effective management and conservation, and that many studies (e.g. movement ecology) require sex determination while keeping animals alive, there is a clear need for non‐lethal, minimally invasive tools for accurate sex identification in this species.

The development of minimally invasive methodologies for sex identification is vital for a host of welfare, conservation and management reasons, including population viability analysis, animal behavioural ecology, remote population monitoring, *ex situ* conservation including captive breeding and veterinary medicine (Ginsberg & Milner‐Gulland, 1994; Holleley et al., 2023; Laopichienpong et al., 2017; Purwaningrum et al., 2019). Tissue biopsy and ultrasonography are two minimally invasive sampling methods that are now commonplace within wildlife research, conservation and management across a range of species (Carroll et al., 2018; Hildebrandt et al., 1995; Turcu et al., 2023; Whittamore et al., 2010). Ultrasonography has been applied to accurately differentiate ovaries and testes in river‐caught wild European eel, although hormonal induction of maturation over an 11 week period was necessary to assess the gonads (du Bureau Colombier et al., 2015). Kucharczyk et al. (2016) successfully used ultrasonography to identify ovaries in European eels without hormone induction. Due to lack of development, the testes were practically invisible on ultrasound, which allowed for accurate sex identification of males by deduction. However, the sample size in this previous study was relatively small (*n* = 10) and these animals were caught in the marine stage of their migration, thus may be more developed than during their riverine stage, making the comparison between ovaries and testes easier. As such, it is not known if ultrasonography is viable, without hormone induction of maturation, for sex identification of European eels during the riverine, freshwater start of their migration, which is the point at which a substantial amount of adult eel capture occurs for monitoring, research or commercial fisheries. A further consideration is that both of these studies used ultrasonography machines suited for laboratory‐based analysis (M‐TurboTM Ultrasound System; SonoSite, Inc.), which are cumbersome for field‐based research. Handheld ultrasound devices have recently been developed (Jung et al., 2021) that have the potential to aid field‐based sex identification in wildlife and have been used in elasmobranchs (Froman et al., 2023). However, to date, the efficacy of these units for sex identification has not been tested in anguillid eels.

Molecular markers from tissue biopsy may also offer potential for sexual identification in European eels (Geffroy et al., 2016). Although morphological differences between sexes in European eels are difficult to differentiate, sexually dimorphic genes may exist in body tissues that could be used as molecular markers for sex identification (Dirks et al., 2014; Hsu et al., 2023). Molecular markers are heritable and observable variants of genes, adaptive or neutral, that can be used to identify genetic variations and associated traits (Gopikrishna, 2023; Kirk & Freeland, 2011). In species with environmental sex determinism, assessing the expression of genes or gene products as molecular markers, rather than sequence differences, can be used to identify sex (Holleley et al., 2023). Recently, the expression level of sexual dimorphic genes from pectoral fin clips in the Japanese eel (*Anguilla japonica*) has been used to identify sex (Hsu et al., 2023), but this methodology has yet to be tested on European eels or other anguillid species.

As the European eel is currently listed as Critically Endangered (Pike et al., 2020), conservation and management measures are in place that could be greatly informed by additional data on sex in this species (Righton et al., 2021). Due to the urgent need for minimally invasive methodologies for sex identification in this species, the aims of this study were to (1) evaluate the use of ultrasonography for sex identification in European eel with both standard and handheld units, and (2) assess whether molecular markers found in Japanese eels can be used to identify sex in European eels.

## 2 MATERIALS AND METHODS

### 2.1 Ethics Statement

The care and use of experimental animals complied with UK animal welfare laws, guidelines and policies. All procedures were subject to ethical approval by the Zoological Society of London Ethics Committee and undertaken within licenced animal facilities (Establishment Licence XBABDAACB) by licenced animal technicians in conjunction with registered zoo veterinary surgeons with experience in fish medicine.

### 2.2 Sample collection

Forty‐seven silver European eels were sampled from the River Haven in southern Lincolnshire, UK, by a commercial eel fisher. Captured eels were transported to the research facility in aerated river water and held in enclosed aerated tanks (500 L) of dechlorinated tap water maintained at 17 ± 1°C using a heater, cooler and temperature control system (D‐D Dual Temp Controller) (Piper et al., 2023).

### 2.3 Euthanasia and sampling

All eels were euthanised by a four‐stage euthanasia procedure, using Home Office Schedule 1 procedures (https://www.legislation.gov.uk/ukpga/1986/14). First, eels were anaesthetised in a bath (20 L) of dechlorinated water and tricaine methanesulfonate solution (MS‐222; 1.5 g/L; Pharmaq Ltd.), buffered with bicarbonate of soda, for a minimum of 30 min and only removed when they had lost all external signs of movement, respiration and response to touch. On removal from the bath, the presence of a heartbeat in each eel was checked for using a GE Versana Active (General Electric Medical Systems) ultrasound machine. Despite the very high dose of MS‐222, all eels had ongoing rhythmic cardiac contractions, regardless of the period of immersion. Once the eels were determined to have reached a deep plane of anaesthesia (loss of righting reflex and no appreciable response to pinching of a pectoral fin and tail), 200 mg/kg of pentobarbital sodium (Pentoject 20% solution, Animalcare Limited) was administered intracardiac with a 25G hypodermic needle. Following the cessation of rhythmic cardiac contractions, the spinal cord was severed with an incision between the skull and first vertebra. Finally, the eel was pithed via insertion of a needle into the brain cavity via the exposed foramen magnum.

Immediately following euthanasia, eels were measured (total body length, *L*_T,_ mm) and weighed (mass, g). A photograph of the left side of each individual was taken to calculate the ocular index (Pankhurst, 1982) and fin index (Durif et al., 2009), both indicators of migratory stage. Horizontal (*D*_h_) and vertical (*D*_v_) eye diameter (mm) and pectoral fin length *L*_F_ (mm) were measured from the photographs using Image J scientific image processing software (https://imagej.net/) and used to calculate the ocular and fin indices from the equations 100 *πL*_T_^−1^(0·25[*D*_h_ + *D*_v_])^2^ and 100 *L*_F_ *L*_T_^−1^, respectively. The entire left and right pectoral fins were collected immediately following measurements, within 2 min of euthanasia, stored on dry ice then either transferred to a −80°C freezer (left pectoral fin) or stored in 1 mL of RNAlater (Ambion) at −20°C (right pectoral fin).

### 2.4 Ultrasonography

**FIGURE 1:**
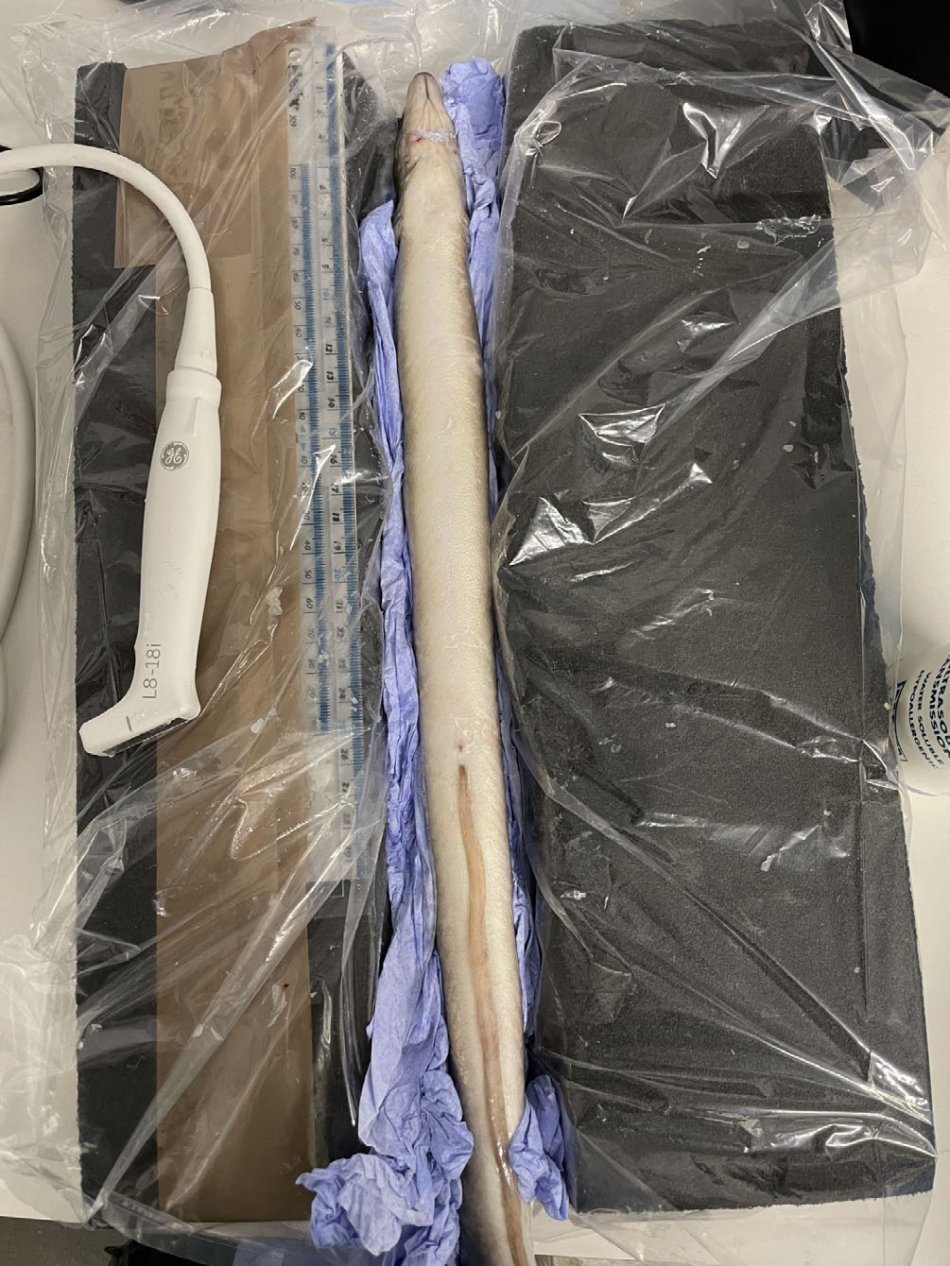
An euthanised European eel (*Anguilla anguilla*). Eels were placed for ultrasound examination in a plastic‐lined foam cradle, padded with paper towel (blue) to minimise slippage. A GE Versana Active probe is shown.

Ultrasound examinations of the eels were performed immediately post‐euthanasia following pectoral fin sampling. Individuals were placed in dorsal recumbency in a linear plastic‐lined foam cradle (Figure 1). A centimetre ruler was taped to the cradle, level with the snout. Eels were scanned along their ventral surface, in the transverse plane (cross‐sectional). Transducer surfaces were covered with acoustic gel to optimise transmission.

**FIGURE 2:**
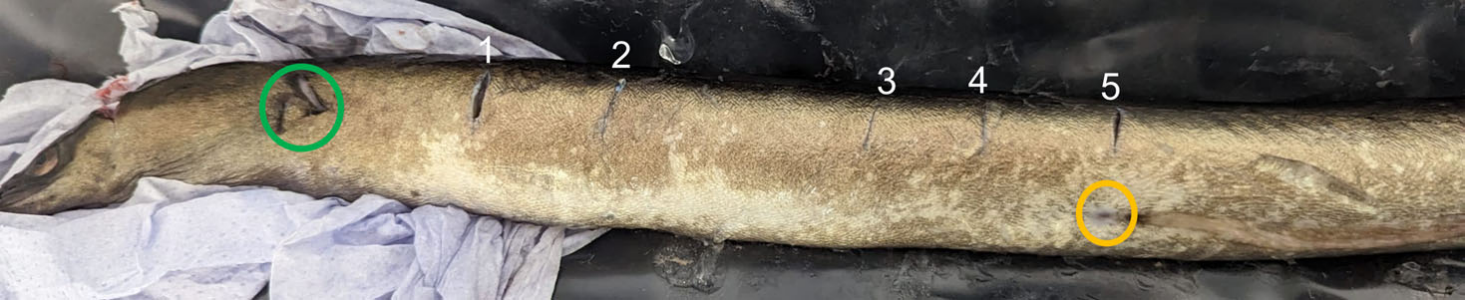
Image of a euthanised European eel (*Anguilla anguilla*) with the five ultrasound scanning sites (1 to 5). The pectoral fin and gill vent is highlighted with a green circle. The anal pore is highlighted with a yellow circle. 1, liver/gall bladder where ovary is first apparent in females; 2, 10–20 mm caudal to site 1, still on the liver and where both ovaries are apparent in females; 3, mid coelom where the swim bladder is first apparent; 4, 10–20 mm cranial to anal pore; 5, anal pore.

Two types of ultrasound unit were used: the GE Versana Active (General Electric Medical Systems), with a the L8‐18i‐RS ‘hockey stick’ probe at 16 MHz and a depth of 5–30 mm, and the GE VScan Air (General Electric Medical Systems) using the Linear 3–12 MHz probe set for ‘vascular’ scanning at 5–30 mm depth (Supporting Information S1 and Table S1). The former is a wired, portable, laptop style unit typically used in a laboratory or medical room setting, whereas the latter is a wireless, handheld unit. For each eel, one of three ultrasonography protocols was used: ‘comprehensive’, ‘full body’ or ‘rapid’ (Table 1). The ‘comprehensive’ and ‘full body’ methods were tested with both the Versana Active and VScan Air units whereas the ‘rapid’ method only used the VScan Air unit. In the ‘full body’ sexing protocol, eels were scanned from pectoral fins to anal pore with five particular locations reviewed based on previous work by Willemse (1979) and Kucharczyk et al. (2016) (Figure 2). These locations were chosen because they can be easily identified using external anatomical structures and thus could be reliably replicated in the field by biologists not trained in ultrasonography. Based on the descriptions by du Bureau Colombier et al. (2015) and Kucharczyk et al. (2016), eels were classified as female if the gonads were easily visualised on ultrasound from the level of the liver and gall bladder continuously through to the anal pore; eels were classified as male, by deduction, if it was not possible to visualise gonads on ultrasound.

**TABLE 1:** A comparison of ultrasonography protocols used for sex identification in the European eel, *Anguilla anguillai* (morphometric data (length, mass) are included).

	Comprehensive protocol	Full‐body protocol	Rapid protocol
Objective	To orientate and familiarise clinicians with the normal ultrasonographic appearance of eels. Undertaken before performing the ‘full body’ and ‘rapid’ protocols.	The eel was scanned from the liver to the urogenital pore to assess the gonads along the full body length.	To assess whether a single‐scan window could be consistently used to sex eels, for potential field use by lay‐people not trained in ultrasonography.
Ultrasound system used	GE Versana Active portable ultrasound + VScan Air	GE Versana Active portable ultrasound + VScan Air	GE VScan Air
Scanning window	Whole body	Pectoral fins (approximate location of heart and gills) to the anal pore with five specific locations reviewed, as per Figure 1.	Single window, 10–20 mm cranial to the anal pore (4, Figure 2).
Number of clinicians performing ultrasounds	One per system, rotated between three.	One per system, rotated between three.	One
Organ identification	Identification of coelomic organs: heart, liver, gall bladder; stomach, anterior GIT; swim bladder; posterior GIT; rectum and anal pore opening, kidney, coelomic fat; gonads. Gonads observed: yes (classified as female); no (classified as male).	Gonads observed: yes (classified as female); no (classified as male).	Gonads observed: yes (classified as female);no (classified as male).
Sample size	6	20	21
Mean length (SD) (mm)	484.0 (131.4)	491.3 (123.4)	457.3 (88.2)
Mean mass (SD) (g)	238.6 (195.5)	237.8 (196.1)	171.7 (97.1)
Additional notes	At the conclusion of the ‘comprehensive’ ultrasounds, the scanning clinician observed the post‐mortem examination to assist with correlation of ultrasound findings to gross anatomy.		

Abbreviations: GIT, gastrointestinal tract; SD, standard deviation.

Initially, six animals were scanned with the ‘comprehensive’ protocol with both the Versana Active and VScan Air units to familiarise the scanning technicians with the normal ultrasonographic appearance of eels. Subsequently, 20 animals were scanned with both the Versana Active and VScan Air units using the ‘full body’ protocol and 21 animals with the VScan Air using the ‘rapid’ protocol (Table 1). At the conclusion of the ultrasound procedure, eels were subsequently stored on ice until post‐mortem examination (maximum 1 h).

### 2.5 Post‐mortem examination and histology

Eels were opened along the ventral midline and the coelomic organs exposed. Major organs (brain, eye, gill, liver, kidney, spleen, gastrointestinal tract and swim bladder) were grossly examined and abnormalities noted. Gonads were removed and sections, no more than 5 mm thick, were fixed in at least 10× volume of 10% neutral‐buffered formalin (Genta Medical) at ambient temperature. The formalin‐fixed gonad was then paraffin embedded and processed into haematoxylin and eosin (H&E) stained sections for histology by standard techniques and examined by a board‐certified pathologist to determine sex. Additionally, one suspected male and one suspected female were used for whole‐body transverse sectioning. These animals were euthanised as above, decapitated and the otoliths and skeletal muscle collected for samples and stored/fixed. Small incisions were made at the cranial and caudal ends of the coelom and a syringe was used to flush the coelom with formalin; the whole carcass was then fixed in a vast excess of formalin. After fixation was complete, the carcasses were decalcified in 10% formic acid and transversely sectioned at five marked locations where ultrasound examinations had been performed (Figure 1). These sections were photographed, then paraffin embedded and processed to H&E sections by standard techniques; the resultant slides were digitised at 20× magnification by using an Axio Scan Z1 (ZEISS) and were read digitally using NDP View 2 (Hamamatsu Photonics).

### 2.6 Molecular markers for sex identification

The expression of three genes (*dcn, LOC111853410* and *kera*) were tested; these were previously identified as having differential expression between male and female Japanese eels, and used successfully to identify sex in this species (Hsu et al., 2023). To test these genes on our European eel samples we directly followed the methodology from Hsu et al. (2023). The *dcn* and *kera* genes encode for decorin precursor‐like protein and keratocan, respectively (Hsu et al., 2023). Decorin plays a role in collagen formation (Chen et al., 2025; Reed & Iozzo, 2002), while keratocan plays a role in corneal development and transparency (Alanazi et al., 2015; Yeh et al., 2008). LOC111853410 encodes a MUC19‐like protein (Hsu et al., 2023), a secreted mucin that is found in numerous tissues in fish, such as the skin, gills and the gastrointestinal tract (Bai et al., 2020; Sharba et al., 2022). An initial exploration of expression values across 13 animals was undertaken (Supporting Information S1 and Table S2), with one additional female animal used as a reference sample. Fin tissue (3.3–27.8 mg) was subsampled from −80°C frozen samples (left pectoral fin) and total RNA extracted using TRIzol reagent (Invitrogen) following the standard protocol then eluted in 30 μL of RNase‐free water. Total RNA was quantified using the broad‐range RNA Quantification kit on a Qubit fluorometer (Invitrogen). First‐strand cDNA synthesis was completed using iScript™ cDNA Synthesis Kit (Bio‐Rad) following the standard protocol for a 20 μL reaction, using 5–10 μL of template, where possible adding between 500 and 1000 ng of RNA.

Quantitative polymerase chain reactions were run on an Applied Biosystems SteOnePlus system. Samples were run in duplicate for the purpose of testing. Samples were tested undiluted and 1:10 diluted. Undiluted samples proved more consistent with a more reasonable CT value (Supporting Information S1 and Table S2). To mitigate variability between qPCR runs data for each gene were collected from a single run, where all samples and the female reference were run in duplicate for the target gene and the housekeeping gene (Supporting Information S1 and Table S3). Samples were run using Meridian Bioscience SensiFAST™ SYBR® Hi‐ROX master mix. Each 10 μL reaction consisted of 0.4 μM of each primer and 1 μL of template cDNA. Cycling conditions consisted of an initial activation hold for 2 min at 95°C followed by 40 cycles of 95°C for 5 s, 60°C for 10 s and 72°C for 20 s, followed by a melt curve analysis.

Replicates were checked for consistent amplification and a mean value calculated. Following methods used in Hsu et al. (2023), the cycle threshold (*C*_T_) value for acidic ribosomal phosphoprotein P0 (*arp*), the housekeeping gene, was subtracted from the average *C*_T_ value of each target for each sample (Supporting Information S1 and Table S4) to get the Δ*C*_T_ value. *arp* was chosen as it is consistently expressed at the same level in eels. Next, for each gene, the Δ*C*_T_ value for a known female reference sample was subtracted from the Δ*C*_T_ values for unknown samples to get the ΔΔ*C*_T_ value. The relative expression levels were calculated as $2^{-\left(∆∆C_{T}\right)}$ for each individual. These were combined with sex identification results from histology to calculate maximum and minimum relative expression levels for each gene across the remaining 13 animals. To assess if there were sex differences in gene expression, Student's *t*‐test (R Core Team, 2024) was used for each gene. If relative expression levels for each gene showed no overlap between male and female eels, or significant differences in expression was seen, relative expression levels were calculated for the rest of the samples. If overlap, or no significant difference, was seen, this indicated there was no sexual differential expression of these genes and further analysis was not undertaken.

PCR products of all genes used in this study (*dcn 99BP*, *LOC111853410 88BP*, *kera 174BP* and *arp 66BP*) were sequenced and found to align with both with the corresponding genes used by Hsu et al. (2023) in Japanese eels and sequences in European eels obtained from GenBank (https://www.ncbi.nlm.nih.gov/genbank/). The four genes matched between 96.59% (3 BP difference) and 100% (0 BP difference) against available sequences in Japanese eel. In addition, all four genes had a 100% match with GenBank sequences of the same genes in European eels.

### 2.7 Statistical analyses

All analyses were undertaken in R Version 4.4.2 (R Core Team, 2024). Analysis of variance (ANOVA) was used to evaluate differences in length and mass between protocol groups. To assess agreement of molecular markers and ultrasound units to identify sex against histological assessment, Cohen's kappa was calculated (Hsu & Field, 2003; McHugh, 2012) using the ‘kappa2’ function from the *irr* package (Gamer & Lemon, 2019).

## 3 RESULTS

Full results from all three protocols and both ultrasound units are presented in Table 2.

**TABLE 2:** Sex identification of European eels (*Anguilla anguilla*) from histology results and three ultrasound scanning methodologies.

Length (mm)	Mass (g)	Ocular index	Fin index	Histological determination of sex	Ultrasound determination of sex using the three different protocols described in Table 1 and two different ultrasound systems						Eel ID
					Comprehensive protocol		Full body protocol		Rapid protocol		
					Versana	VScan air	Versana	VScan air	Versana	VScan air	
333	56.1	6.87	5.66	M	–	–	M	M	–	–	XT060/24
344	64.4	5.21	5.21	M	–	–	–	–	–	M	XT757/23
348	78.7	7.41	4.09	M	–	–	M	M	–	–	XT739/23
350	64.6	5.45	4.7	M	–	–	–	–	–	M	XT748/23
351	68.5	7.04	5.8	M	–	–	–	–	–	M	XT760/23
355	90.9	6.17	6.14	U	–	–	M	M	–	–	XT733/23
356	74.1	7.38	4.68	M	M	M	–	–	–	–	XT723/23
360	86.3	5.62	4.99	M	M	M	–	–	–	–	XT724/23
367	89	7.46	5.21	M	–	–	–	–	–	M	XT761/23
367	89.8	8.49	5.53	M	–	–	–	–	–	M	XT763/23
373	92.4	7.65	5.56	U	–	–	–	–	–	M	XT759/23
376	98.9	8.59	5.22	M	–	–	M	M	–	–	XT728/23
381	69.4	6.87	5	M	–	–	M	M	–	–	XT738/23
388	92.5	6.84	4.76	M	–	–	–	–	–	M	XT751/23
391	105.2	7.37	5.23	M	M	M	–	–	–	–	XT726/23
392	103.8	5.9	4.94	M	–	–	M	M	–	–	XT729/23
394	93.7	5.69	4.68	M	–	–	–	–	–	M	XT762/23
397	103.2	6.68	5.48	U	–	–	M	M	–	–	XT731/23
399	103.1	7.43	5.41	M	–	–	–	–	–	M	XT753/23
407	93.9	3.92	5.64	U	–	–	M	M	–	–	XT737/23
424	116.6	5.46	5.08	M	–	–	M	M	–	–	XT741/23
436	134.4	6.05	5.31	M	–	–	–	–	–	M	XT750/23
441	131.4	7.43	4.21	M	–	–	–	–	–	M	XT765/23
458	160.9	7.49	5.29	M	–	–	–	–	–	M	XT764/23
484	143.7	6.22	5.13	F	–	–	–	–	–	F	XT752/23
499	210.5	5.87	5.21	F	–	–	F	F	–	–	XT732/23
502	215.7	7.01	5.68	F	–	–	–	–	–	F	XT758/23
529	229.3	6.52	5.24	F	–	–	F	F	–	–	XT735/23
532	256.6	7.65	5.69	F	–	–	F	F	–	–	XT743/23
535	264.6	8.4	5.07	F	–	–	F	M	–	–	XT730/23
535	277.4	8.68	5.73	F	–	–	F	F	–	–	XT734/23
537	237.5	5.19	4.83	F	F	F	–	–	–	–	XT721/23
545	266.3	4.66	4.57	F	–	–	F	F	–	–	XT744/23
547	294.4	5.93	5.28	F	–	–	–	–	–	F	XT747/23
548	316.4	7.33	5.33	F	–	–	F	F	–	–	XT742/23
551	253.3	5.45	5.15	F	–	–	–	–	–	F	XT756/23
563	282.5	7.54	6.71	F	–	–	–	–	–	F	XT749/23
564	311.2	5.34	4.74	F	–	–	–	–	–	F	XT755/23
568	255.6	7.71	5.51	F	–	–	–	–	–	F	XT745/23
578	343	5.11	5.1	F	–	–	–	–	–	F	XT754/23
579	321.3	6.21	4.31	F	–	–	–	–	–	F	XT746/23
592	336	7.79	5.73	F	–	–	F	F	–	–	XT059/24
607	332.9	5.6	4.83	F	–	–	F	F	–	–	XT736/23
614	361.4	7.93	5.04	F	F	F	–	–	–	–	XT725/23
646	567.2	7.89	5.13	F	F	F	–	–	–	–	XT722/23
705	620.3	7.48	3.81	F	–	–	F	F	–	–	XT740/23
786	834.9	10.44	4.83	F	–	–	F	F	–	–	XT727/23

*Note*: Eel ID, length, ocular index and pectoral fin index are also presented. For histological determination of sex, eels were classified as either male (M), female (F) or undetermined sample (U). For ultrasound determination of sex, eels were classified as female (F) if gonads were identified and male (M) if no gonads were identified. –, this method of sex determination was not undertaken on this animal.

**FIGURE 3:**
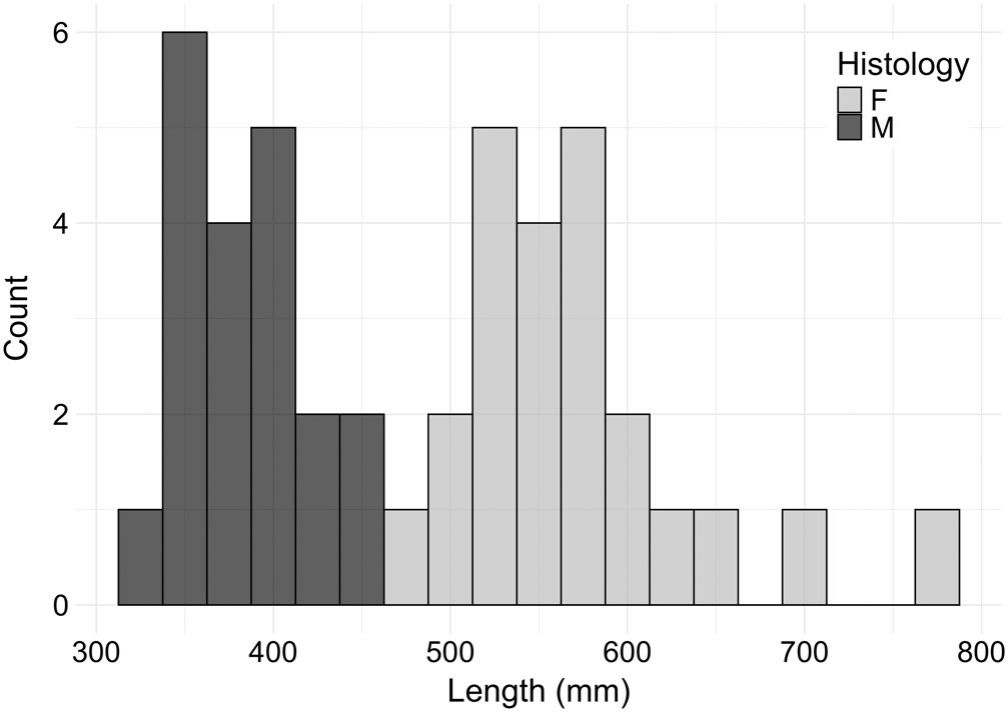
Histogram of eel lengths (mm) by sex. Male lengths are indicated by black bars and female lengths by grey bars.

ANOVA tests indicated there were no significant differences in the lengths (*F*_2, 44_ = 0.51, *p* value = 0.60) or mass (*F*_2, 44_ = 1.0, *p* value = 0.30) of eels between the three protocols. A histogram of eel lengths in the study is presented in Figure 3.

### 3.1 Dissection and histology

Female gonads were readily distinguishable as linear arrangements of frond‐like, white tissue originating bilateral to the swim bladder and extending into the ventral coelom, with an approximate cross‐sectional diameter of 10–20 mm. They were a similar colour and texture to fat but did not float in formalin or water. Male gonads were similarly linear and bilateral to the swim bladder, but were much thinner, appearing as smooth, white fibres less than 1 mm in diameter. Sex could be identified in 91% (43/47) of European eels using histological examination. Of the 47 animals sampled 49% (*n* = 23) were female, 42% male (*n* = 20) and 9% (*n* = 4) did not contain enough sample to enable assessment (Table 2). Of the 43 animals where sex could be determined by histology, all animals less than 460 mm were male (*n* = 16) and all animals greater than 500 mm were female (*n* = 21). Of those 43 animals, six individuals had lengths between 400 and 500 mm. Two were female and four were male. Every sex identified histologically was also identified grossly and the four unidentified samples were called as males grossly due to lack of visible gonad.

### 3.2 Ocular and fin indices

Ocular and fin indices ranged from 3.92 to 10.44 (mean = 6.78 ± 1.24 SD) and 3.81 to 6.71 (mean = 5.16 ± 0.52 SD), respectively, with 59.6% (28/47) of eels having an ocular index greater than 6.5 and therefore classed as sexually mature adults (Pankhurst, 1982). In the 23 animals that were designated female from histology, 96% (22/23) had a fin index greater than 4.3, indicating they were at the silver migratory stage (Durif et al., 2009). In the 20 animals that were designated male from histology, 75% (15/20) had a fin index greater than 4.7, indicating they were at the silver migratory stage (Durif et al., 2009).

### 3.3 Molecular marker assessment

**FIGURE 4:**
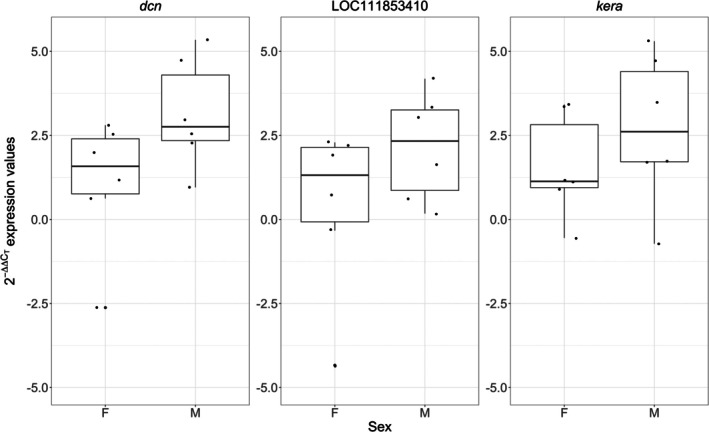
Boxplots of the distribution of $2^{-\left(∆∆C_{T}\right)}$ expression values of *dcn*, *LOC111853410* and *kera* genes, in fin material of riverine stage European eels (*Anguilla anguilla*) by sex, normalised to a housekeeeping gene (*arp*) and using a female sample (XT721/23) as an internal control to calculate relative expression levels, *n* = 13.

From an initial sample of 13 individuals, expression values, $2^{-\left(∆∆C_{T}\right)}$, showed overlap between the sexes in all three genes (Figure 4, Supporting Information S1 and Table S3), with no significant differences in expression values between the sexes for *dcn* (*t* [9.63] = −1.95, *p* = 0.08), *LOC111853410* (*t* [8.45] = −1.42, *p* = 0.19) or *kera* (*t* [8.9] = −1.02, *p* = 0.33). This overlap, and no significant difference between the sexes across all three genes, suggests that these genes cannot be used to reliably differentiate sex in European eels, and, as such, further analysis was not conducted on the rest of the samples. RNA Integrity Number scores varied from 7.2 to 8.9.

### 3.4 Ultrasonography

**FIGURE 5:**
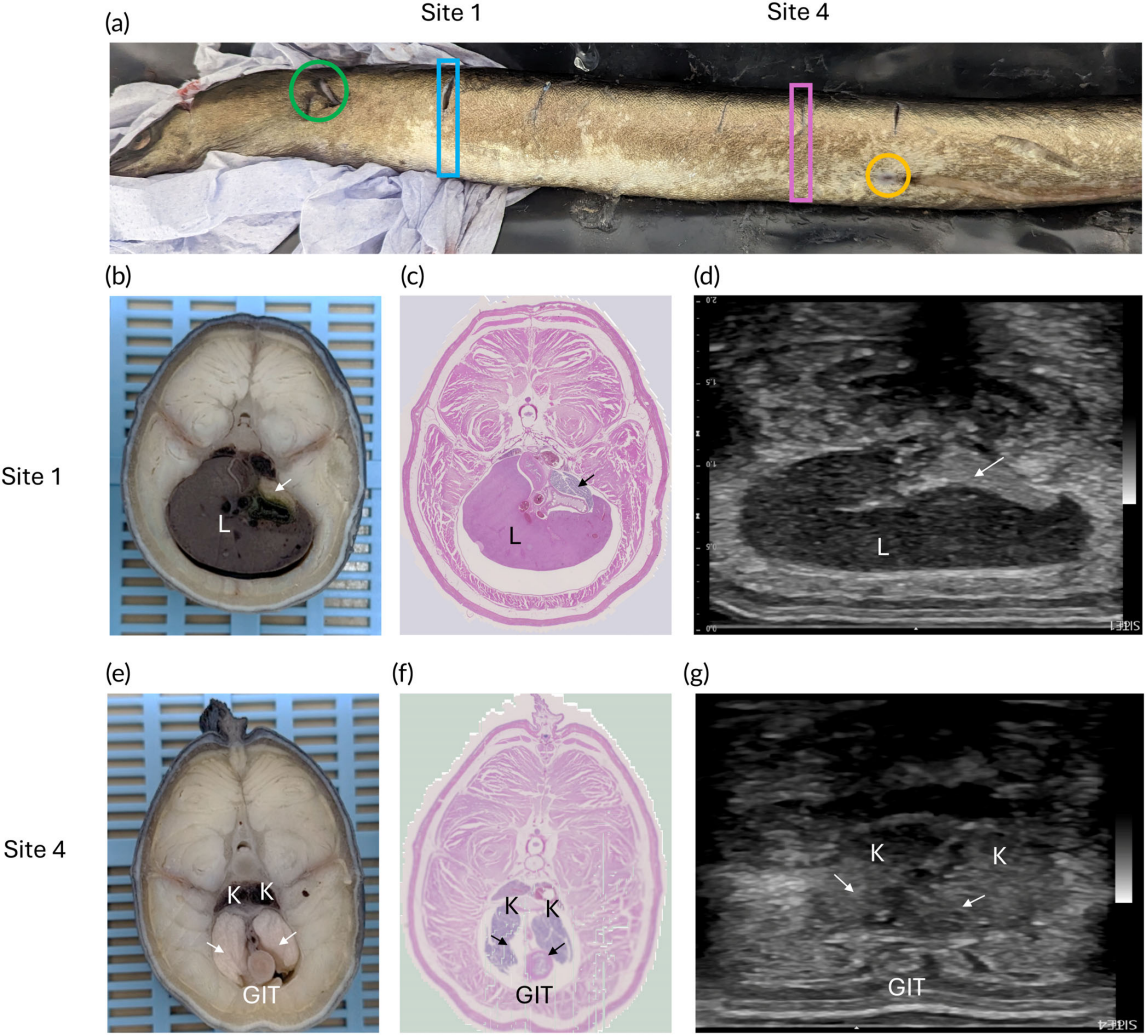
Fixed, histological and ultrasound images of female European eels (*Anguilla anguilla*). (a) Image of a female European eel (*Anguilla anguilla*, XT059/24) and location of scan sites 1 and 4. Scan site 1 is indicated by a blue rectangle and scan site 4 is indicated by a pink rectangle. The pectoral fin and gill vent are indicated by a green circle and the anal pore by a yellow circle. Fixed section (b), histological section (c) and Versana ultrasound image (d) at scan site 1. Fixed section (e), histological section (f) and Versana ultrasound image (g) at scan site 4. Locations of the liver (L), gastrointestinal tract (GIT) and kidneys (K) are indicated, with the presence of gonad indicated by white or black arrows.

**FIGURE 6:**
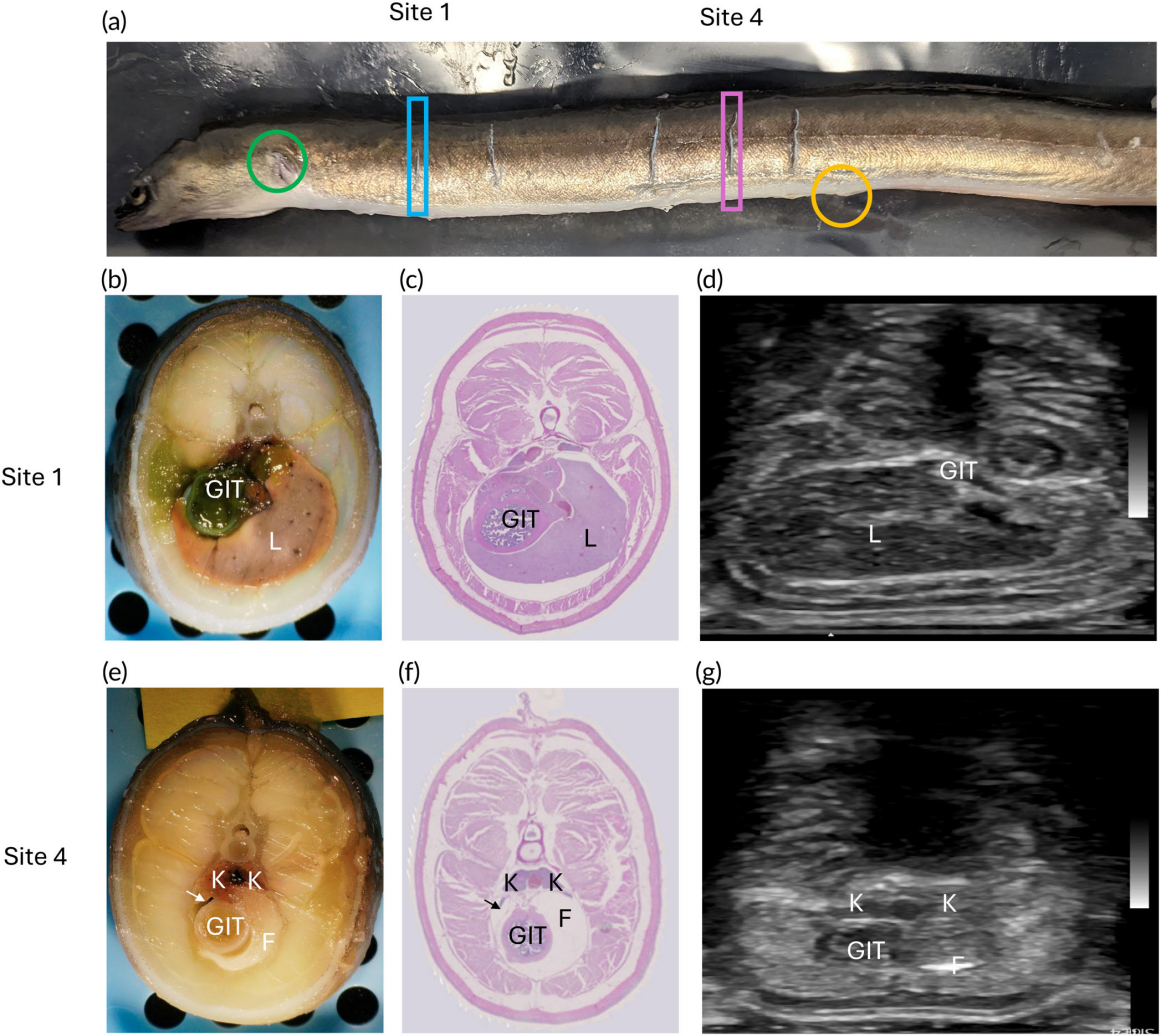
Fixed, histological and ultrasound images of male European eels (*Anguilla anguilla*). (a) Image of a male European eel (*Anguilla anguilla*, XT060/24) and location of scan sites 1 and 4. Scan site 1 is indicated by a blue rectangle and scan site 4 is indicated by a pink rectangle. The pectoral fin and gill vent are indicated by a green circle and the anal pore by a yellow circle. Fixed section (b), histological section (c) and Versana ultrasound image (d) at site 1 are shown. Fixed section (e), histological section (f) and Versana ultrasound image (g) at site 4 are also show. Locations of gastrointestinal tract (GIT), fat (F), kidney (K) and liver (L) are indicated. No gonad visible in any image, indicating that the individual is male.

Animals were classified as female if gonads were visualised on ultrasound (Supporting Information S2 and S3). Using the ‘comprehensive’ and ‘full body’ protocols, which started scanning at the pectoral fins, the female gonads were identifiable as hypoechoic structures against the denser hepatic tissue, with the right ovary appearing just cranial to the gall bladder (scan site 1, Figure 2; Figure 5), and the left ovary slightly more caudal. Both could be followed continuously through to the anal pore (scan site 5, Figure 2). Eels were classified as male by deduction if it was not possible to visualise the gonads using ultrasound (Figure 6). Both protocols and both systems of ultrasound equipment allowed visualisation of the gonads when they were visible. Using the ‘rapid’ protocol (single window 10–20 mm cranial to the anal pore [scan site 4, Figure 2; wireless handheld VScan Air system]) allowed a view of the caudal gastrointestinal tract with female gonads visible as paired, hypoechoic, triangular structures ventral to the denser renal tissue and dorsal to the gastrointestinal tract. Examples of ultrasound images along with matched images from histology and fixed sections are presented in Figures 5 and 6 and Supporting Information S2 and S3.

Sex was designated in all 47 eel ultrasounded (Table 2). Of the 43 animals which had histological sex confirmation, and therefore where comparison was possible, six were scanned with the ‘comprehensive’ protocol (both units), 17 with the ‘full body’ protocol (both units) and 20 with the ‘rapid’ protocol (VScan Air) (Tables 1 and 2). All (23/23) of the animals scanned using the Versana unit matched histological sex identification and 98% (42/43) of animals scanned with the VScan Air unit matched histological sex identification. Cohen's kappa values, assessing agreement of ultrasound units to identify sex against histological assessment, were between 0.88 and 1.00 for all comparisons (Table 3), indicating excellent agreement between all ultrasound units and histology.

**TABLE 3:** Cohen's kappa values of ultrasound protocols compared to histological sex identification in European eels (*Anguilla anguilla*).

Histology comparison	Ultrasound unit	Cohen's kappa	Z value	*p* value
Comprehensive	Versana	1.00	2.45	0.01
Full body	Versana	1.00	4.12	<0.001
Comprehensive	VScan Air	1.00	2.45	0.01
Full body	VScan Air	0.88	3.64	<0.001
Rapid	VScan Air	1.00	4.47	<0.001

*Note*: Z value and p values are presented.

Seven animals had lengths between 400 and 500 mm, which is the size range most associated with ambiguity in sex identification using body morphology alone (du Bureau Colombier et al., 2015; Tesch, 2003). Six of these had histological sex identification, which matched 100% with the ultrasound sex identification (Table 2).

## 4 DISCUSSION

The European eel is currently listed as Critically Endangered by the IUCN (Pike et al., 2020). As such, significant conservation and management actions are required to restore populations of this species. For management actions to be effective, information on the biology and ecology of European eels, such as differential behaviour between sexes, is required. However, to date, no accurate, non‐lethal methodology of assessing sex in European eels has been available. Here, sex identification using the portable Versana unit matched histological sex identification in 100% (23/23) of subjects and the handheld VScan Air unit matched 98% (42/43). Therefore, we show that two types of ultrasound unit can be used to accurately identify sex in silver riverine European eels, without requiring fish sacrifice or hormone induction of maturation. However, we found that three molecular markers previously used to accurately differentiate sex in Japanese eels cannot be used reliably to identify sex in European eels. Ultrasound methods, including a portable handheld device suitable for in‐field sex identification, therefore offer accurate, minimally invasive and reliable alternatives to current assessment methods for sex identification in European eels, particularly in eels at intermediate sizes and animals in habitats where migration potential is limited and may regularly exceed typical growth sizes. This presents an opportunity to further our knowledge of the behaviour and ecology across anguillid eels to aid conservation and management techniques in these species. Our results are consistent with earlier studies that suggest using size to identify sex in silver eels can be unpredictable at certain size ranges, with a mixture of males and females found between 400 and 500 mm.

Our findings support previous work indicating that ultrasonography can be used to identify sex in European eels. du Bureau Colombier et al. (2015) and Müller et al. (2016) previously used ultrasonography to determine sex in European eels exposed to hormone treatment, and Kucharczyk et al. (2016) used ultrasonography to determine sex in late stage migratory eels caught in the Baltic Sea. However, we found that sex identification can also be achieved without hormone induction of maturation and in individuals at the early stage of migration. Sex in eels could be identified using a waterproof handheld ultrasound unit, which could be utilised in the field. Although accuracy for the handheld Vscan unit was at 98% and it was not 100% reliable, unlike the Versana unit, these technologies are constantly improving and the resolution, and therefore accuracy, of the handheld VScan unit should be the same level as the Versana unit in the near future. Further, although sample size was small, ultrasound methods were 100% effective at identifying the sex of European eels at intermediate sizes, suggesting that use of ultrasound may be more effective than using size‐based thresholds in animals between 460 and 500 mm, where sex size thresholds are often unreliable.

In addition, we identified a scanning window that can be reliably and repeatedly used (4, Figures 2, 4 and 5) for sexing. Although site 2 may be slightly clearer for distinguishing gonads from other tissue (Supporting Information S2 and S3), site 4 (10–20 mm cranial to the anal pore) was chosen to see if it would be possible to find a single anatomical area to place the probe that would be useful for untrained or ‘lay’ ultrasonography to be undertaken by biologists not trained in ultrasonography techniques. There is no external landmark for placing the probe for site 2 and it relies on understanding more of the structures being scanned. Being able to rapidly place the probe on site 4 to sex eels also reduces the amount of time spent scanning to keep handling in live animals to a minimum. As such, this site could be located with minimal training, thereby allowing field biologists to identify female gonads when present and identify males by deduction. Gonads at this site were sufficiently visible that we believe only minimal training would be needed to determine sex from the images. This means sex identification could be undertaken rapidly as a part of field studies, reducing animal stress levels and avoiding animals having to be moved into a laboratory setting. This would be particularly useful for experimental or conservation and management work that may require equal sex ratios. It should be noted that ultrasonography requires anaesthesia to be effective, which always has welfare implications, and has the potential to impact behaviour and therefore influence the results of any study (Cooke et al., 2013; Macaulay et al., 2021; Soulsbury et al., 2020). However, many field techniques in eels require anaesthesia as it can reduce stress during handling (Iversen et al., 2013; Walsh & Pease, 2002), and handheld ultrasonography could be simply integrated into these processes. As such, this ultrasonography is most practical for biologists during these scenarios, and would not necessarily justify the use of anaesthesia solely for the process of sex identification. However, this method should be tested with anaesthetised eels during other in‐field methodologies as a next step.

Previous work has also found that male gonads in European eels were practically invisible with ultrasonography, and thus, through deduction, allowed for practically error‐free sex identification (du Bureau Colombier et al., 2015; Kucharczyk et al., 2016). Although sex identification by visible classification of gonads may be preferable, identification of sex through deduction is not uncommon in fish and has been undertaken in Atlantic salmon (*Salmo salar*) (Mattson, 1991), Murray cod (*Maccullochella peelii peelii*) (Newman et al., 2008), striped bass (*Morone saxatilis*) (Blythe et al., 1994) and sturgeon (*Acipenser stellatus*) (Moghim et al., 2002). Using this method, this study found that sex could be correctly identified in between 98% and 100% of animals using ultrasonography in the current study (depending on the unit used), but only 91% of animals using histological assessment. Histology was an imperfect method for sex identification because, on some occasions, gonad tissue was too small to sample or could not be taken.

The eels sampled in our study had clear length cut‐offs, with all animals less than 460 mm identified as male. However, our sample size was small and two females had lengths of less than 500 mm. Data from previous studies, with larger sample sizes, have suggested that using size‐based criteria for sex ID may not always be accurate. Sex identification in European eels based on body morphology has previously been found to be uncertain within the size range of 400–500 mm (du Bureau Colombier et al., 2015; Tesch, 2003). We found similar results with a mix of sexes identified among the seven animals within this range. Male eels raised from glass eels to silver in captivity have reached sizes of 580 mm (Beullens et al., 1997a) and research on the lengths of 767 silver European eels from six countries found females ranged from 406 to 1005 mm and males from 288 to 512 mm (Boulenger et al., 2015). Two recent studies have found a female silver eel range from 418 to 634 mm (Hala et al., 2025) and 357–635 mm (Milošević et al., 2022). As such, these reported size ranges indicate that this range of uncertain lengths for sex ID may be broader or more variable among regions than previously suggested. Where paired histology and ultrasonography were performed, ultrasonography had 100% accuracy with the portable Versana unit and 98% accuracy with the handheld Vscan Air unit.

Given that histological assessment requires fish sacrifice and, in this study, had a lower probability of sex identification, ultrasound should be preferred over sacrificial sampling as a method to identify sex in European eels. In addition, ultrasound could also provide an alternative to size‐based thresholds, particularly at uncertain size ranges. For example, Denis et al. (2022) identified sex in 804 out of 1620 eels (643 females, 161 males) from estuarine habitats in the English Channel using the silvering index method. If size‐based thresholds (<460 mm male, >500 mm female) had been used, although only 0.01% (*n* = 2) males were found to be greater than 500 mm (and therefore misidentified as females), 9% of females had lengths of under 460 mm and would have been misidentified as males. In addition, 17% of animals (*n* = 139) lay in the intermediate zone between 460 and 500 mm and would not have given a sex identification. In the current study, only 60% of animals had an ocular index of greater than 6.5, indicating they were sexually mature; however, sex was identified with ultrasonography in the majority of cases, indicating that ultrasonography can be used to identify sex in animals even before they are fully sexually mature.

Results also showed that the three genes identified as having differential expression between males and females in Japanese eels could not be used to reliably differentiate sex in European eels. Interestingly, this suggests that sexually differentially expressed genes in pectoral fins of anguillid species may not be conserved. It is expected that genes are generally conserved between closely related species (Naqvi et al., 2019; Philipp et al., 1979; Sun et al., 2020; Whitehead & Crawford, 2006), and genes for hormone production (Morini et al., 2015) and larval development (Bernatchez et al., 2011) were previously shown to be conserved between anguillid species. However, sex‐biased genes can evolve rapidly and differences in their gene expression between species and populations can occur (Ellegren & Parsch, 2007). In addition, phylogenetic trees generally separate European and Japanese eels into different clades by geographical region (Aoyama et al., 2001; Minegishi et al., 2005). As such, it may not be surprising that there is differential gene expression between these species and sex‐biased genes are not conserved. Alternatively, it may be that the Japanese eels used by Hsu et al. (2023) were at a later stage of maturation than the animals used here, and as such animals were more developed and relative gene expression between sexes is more likely to be pronounced. However, mean ocular and fin indices were 6.78 and 5.16, and 6.03 and 5.20, in this study and Hsu et al. (2023), respectively, suggesting a similar maturation stage among subjects in the two studies. The average length and mass of animals in Hsu et al. (2023) were larger than the current study, but this is most likely due to size differences between the two species (Righton et al., 2021).

Molecular markers have been used to identify sex in a number of species that have environmental sex determination and/or minimal/late stage sexual dimorphism, such as snakes (Laopichienpong et al., 2017; Rovatsos et al., 2015), lizards (Quinn et al., 2009), alligators (Janes et al., 2013), testudines (Literman et al., 2014) and fish (Komrakova et al., 2018; Li et al., 2020; Yu et al., 2022). However, the efficacy of this technique is often species dependent. For example, molecular methods for sex identification have not been successful in American alligators (*Alligator mississippiensis*) (McCoy et al., 2016), green turtles (*Chelonia mydas*) (Mayne et al., 2023; Sönmez et al., 2019) and Indian garden lizards (*Calotes versicolor*) (Wilson et al., 2019). It may be that the use of molecular markers for sex identification is not feasible in European eels. However, here we tested for three sexually dimorphic genes, identified from pectoral fins in Japanese eel. To comprehensively test the feasibility of sex identification through molecular markers in European eels, future research should follow the full process by Hsu et al. (2023) and identify a suite of candidate sexually dimorphic genes from pectoral fin samples, and assess the expression of all these candidate genes between animals of known sex. In addition, transcriptome comparison from fin tissues may be an effective method for identifying sexually dimorphic genes and has previous been used in fish species (Zhou et al., 2023). Alternatively, sex identification using epigenetics and differences in DNA methylation has recently been carried out in green turtles (*Chelonia mydas*) (Mayne et al., 2023). However, methylation patterns in eels are potentially too condition dependent to be reliably used (Chapelle & Silvestre, 2022). Differential expression of microRNAs have been recently adapted for sex identification in fish species (Houdelet et al., 2023; Zhang et al., 2019) and sex hormones in plasma have been identified in plasma of other anguillid species (Hwang et al., 2022). Finally, genes involved in sexual expression have been found in European eel gonadal tissue (Geffroy et al., 2016). As genes involved in sexual expression have been found in non‐gonadal tissues in European seabass (*Dicentrarchus labrax*), the expression of genes isolated by Geffroy et al. (2016) in fin tissue could be investigated. As such there are several methods to be tested which offer interesting avenues of further research for this burgeoning field.

In this study, we tested sex identification methods in early‐stage, riverine, migratory European eels. However, sex determination in European eels occurs earlier, during the yellow eel stage (Geffroy & Bardonnet, 2016), but is only identifiable from histological samples when animals reach 200–300 mm (Beullens et al., 1997a; Colombo et al., 1984; Colombo & Grandi, 1996). The ecology and behaviour of yellow eels compared to the silver migratory phase is markedly different (Bašić et al., 2019; Lennox et al., 2018; McCleave & Arnold, 1999). Sex identification at this development stage would provide vital ecological information on this species. Female yellow eels do show ovary development (Beullens, Eding, Ollevier, et al., 1997) and as such ultrasound may be capable of identifying early differentiated ovaries at this stage. Although intersex gonads are also present at the yellow stage, and the majority of these differentiate into testes, and therefore males (Beullens et al., 1997a), due to their size, they are unlikely to be detected by ultrasound. Determining if ultrasound, and other minimally invasive methods, can be used to sex the resident, yellow eel development stage should be prioritised as the next step in this field.

Sex identification is important, not only for the basic understanding of the ecology and behaviour of threatened species, but also for establishing and applying conservation and management plans (Laopichienpong et al., 2017). Minimally invasive sex identification tools therefore have the potential to greatly improve ecological knowledge of European eels, and potentially other anguillid species. For example, ultrasonography could be utilised to further ecological knowledge on the drivers of sex determination, which are poorly understood, as well as sex differences in habitat use. Habitat loss and modification are major threats to anguillid eels (Drouineau et al., 2018; Jacoby et al., 2015) and sex identification is fundamental for understanding fine‐scale habitat use, which is a critical component of any conservation and management plan. Sex information on European eels would also enhance other aspects of conservation and management, such as investigating the differential impacts of anthropogenic threats between sexes. For example, it is unknown how the anthropogenic impacts of pollution, climate change and fishing pressure alter sex ratios in European eels (Crowley et al., 2022; Righton et al., 2021; van Ginneken & Niemantsverdriet, 2017). If these anthropogenic pressures result in a skew towards females (typically the sex with the greatest influence on population growth), population impacts may be negligible (Ginsberg & Milner‐Gulland, 1994; Kendall & Quinn, 2013) but could alter sexual selection processes. If these pressures result in relative decreases of females in the population, this may have significant negative impacts on population growth and recruitment (Uusi‐Heikkilä, 2020).

Here, we evaluated two minimally invasive methods for sex identification in European eels and found that two types of ultrasonography unit were effective at identifying sex in this species. However, molecular methods, previously successful for identifying sex in Japanese eels, were unsuccessful in European eels. Ultrasonography, therefore, provides a viable, non‐lethal, alternative to histological assessment, which requires fish sacrifice, alongside an alternative to size‐based sex ID at intermediate sizes and animals in habitats where migration potential is limited and may regularly exceed typical growth sizes. This method has the potential to be used to answer a variety of questions to aid the conservation and management of European eels, as well as discover more about the biology and ecology of this enigmatic species.

## AUTHOR CONTRIBUTIONS

M.J.W.: Conceptualization (equal), data curation (equal), formal analysis (lead), methodology (equal), investigation (equal), visualisation (lead), writing – original draft (lead), writing – review and editing (lead); J.A.B.: Writing – review and editing; K.H.: Conceptualization (equal), methodology (supporting), writing – review and editing (supporting); L.O.: Investigation (supporting), writing – review and editing (supporting); M.W.P.: Investigation (supporting), resources (supporting), writing – review and editing (supporting); C.S.: Investigation (supporting), resources (supporting), writing – review and editing (supporting); S.S.: Investigation (supporting), methodology (supporting), resources (supporting), writing – review and editing (supporting); T.S: Investigation (supporting), methodology (equal), resources (supporting), writing – review and editing (supporting); R.S.W: Writing – review and editing); J.W.: Investigation (supporting), writing – review and editing (supporting); E.W.: Investigation (supporting), writing – review and editing (supporting); R.M.W.: Funding acquisition (equal), resources (supporting), writing – review and editing (supporting); A.T.P.: Conceptualization (equal), funding acquisition (equal), investigation (equal), methodology (equal), resources (leading), supervision (leading), writing – review and editing (supporting).

## FUNDING INFORMATION

This research was principally funded by the Environment Agency. A.T.P. was part‐funded by Research England.

## CONFLICT OF INTEREST STATEMENT

The authors declare that they have no known competing financial interests or personal relationships that could have appeared to influence the work reported in this paper.

## Supporting information


**TABLE S1.** Ultrasound systems used for sex identification in the European eel, *Anguilla anguilla*.
**TABLE S2.** CT values between undiluted and diluted samples for *arp* HK and *dcn* genes.
**TABLE S3.** Eel ID and histological sex identification with 2^ΔΔC^_T_ values for dcn, LOC111853410 and kera genes for a subsample of 14 eels.
**TABLE S4.** CT values for *arp*, LOC111853410, *kera* and *dcn* genes for the initial exploration of expression values across 13 animals.

**DATA S1.** Supporting Information.

**DATA S2.** Supporting Information.

## Data Availability

Raw data supporting the results are available from the Zenondo Digital Repository: https://zenodo.org/records/14871158.

## References

[jfb70361-bib-0001] Adeogun, A. O. , Ibor, O. R. , Omiwole, R. , Chukwuka, A. V. , Adewale, A. H. , Kumuyi, O. , & Arukwe, A. (2020). Sex‐differences in physiological and oxidative stress responses and heavy metals burden in the black jaw tilapia, *Sarotherodon melanotheron* from a tropical freshwater dam (Nigeria). Comparative Biochemistry and Physiology Part C: Toxicology & Pharmacology, 229, 108676. 31783175 10.1016/j.cbpc.2019.108676

[jfb70361-bib-0002] Alacs, E. A. , Georges, A. , FitzSimmons, N. N. , & Robertson, J. (2010). DNA detective: A review of molecular approaches to wildlife forensics. Forensic Science, Medicine, and Pathology, 6, 180–194. 20013321 10.1007/s12024-009-9131-7

[jfb70361-bib-0003] Alanazi, S. A. , Almubrad, T. , AlIbrahim, A. I. A. , Khan, A. A. , & Akhtar, S. (2015). Ultrastructure organization of collagen fibrils and proteoglycans of stingray and shark corneal stroma. Journal of Ophthalmology, 2015, 686914. 26167294 10.1155/2015/686914 PMC4488252

[jfb70361-bib-0004] Aoyama, J. , Nishida, M. , & Tsukamoto, K. (2001). Molecular phylogeny and evolution of the freshwater eel, genus Anguilla. Molecular Phylogenetics and Evolution, 20, 450–459. 11527470 10.1006/mpev.2001.0959

[jfb70361-bib-0005] Bade, A. P. , Binder, T. R. , Faust, M. D. , Vandergoot, C. S. , Hartman, T. J. , Kraus, R. T. , Krueger, C. C. , & Ludsin, S. A. (2019). Sex‐based differences in spawning behavior account for male‐biased harvest in Lake Erie walleye (*Sander vitreus*). Canadian Journal of Fisheries and Aquatic Sciences, 76, 2003–2012.

[jfb70361-bib-0006] Bai, J. , Hu, X. , Lü, A. , Wang, R. , Liu, R. , Sun, J. , & Niu, Y. (2020). Skin transcriptome, tissue distribution of mucin genes and discovery of simple sequence repeats in crucian carp (*Carassius auratus*). Journal of Fish Biology, 97, 1542–1553. 32885862 10.1111/jfb.14524

[jfb70361-bib-0007] Bašić, T. , Aislabie, L. , Ives, M. , Fronkova, L. , Piper, A. , & Walker, A. (2019). Spatial and temporal behavioural patterns of the European eel *Anguilla anguilla* in a lacustrine environment. Aquatic Sciences, 81, 73.

[jfb70361-bib-0008] Bernatchez, L. , St‐Cyr, J. , Normandeau, E. , Maes, G. E. , Als, T. D. , Kalujnaia, S. , Cramb, G. , Castonguay, M. , & Hansen, M. M. (2011). Differential timing of gene expression regulation between leptocephali of the two anguilla eel species in the Sargasso Sea. Ecology and Evolution, 1, 459–467. 22393514 10.1002/ece3.27 PMC3287341

[jfb70361-bib-0009] Bertram, M. G. , Saaristo, M. , Baumgartner, J. B. , Johnstone, C. P. , Allinson, M. , Allinson, G. , & Wong, B. B. M. (2015). Sex in troubled waters: Widespread agricultural contaminant disrupts reproductive behaviour in fish. Hormones and Behavior, 70, 85–91. 25797925 10.1016/j.yhbeh.2015.03.002

[jfb70361-bib-0010] Beullens, K. , Eding, E. H. , Gilson, P. , Ollevier, F. , Komen, J. , & Richter, C. J. J. (1997). Gonadal differentiation, intersexuality and sex ratios of European eel (*Anguilla anguill* L.) maintained in captivity. Aquaculture, 153, 135–150.

[jfb70361-bib-0011] Beullens, K. , Eding, E. H. , Ollevier, F. , Komen, J. , & Richter, C. J. J. (1997). Sex differentiation, changes in length, weight and eye size before and after metamorphosis of European eel (*Anguilla Anguilla* L.) maintained in captivity. Aquaculture, 153, 151–162.

[jfb70361-bib-0012] Blythe, B. , Helfrich, L. A. , Beal, W. E. , Bosworth, B. , & Libey, G. S. (1994). Determination of sex and maturational status of striped bass (*Morone saxatilis*) using ultrasonic imaging. Aquaculture, 125, 175–184.

[jfb70361-bib-0013] Boulenger, C. , Acou, A. , Trancart, T. , Crivelli, A. J. , & Feunteun, E. (2015). Length–weight relationships of the silver European eel, *Anguilla Anguilla* (Linnaeus, 1758), across its geographic range. Journal of Applied Ichthyology, 31, 427–430.

[jfb70361-bib-0014] Burger, J. , Fossi, C. , McClellan‐Green, P. , & Orlando, E. F. (2007). Methodologies, bioindicators, and biomarkers for assessing gender‐related differences in wildlife exposed to environmental chemicals. Environmental Research, 104, 135–152. 17207477 10.1016/j.envres.2006.08.002

[jfb70361-bib-0015] Carroll, E. L. , Bruford, M. W. , DeWoody, J. A. , Leroy, G. , Strand, A. , Waits, L. , & Wang, J. (2018). Genetic and genomic monitoring with minimally invasive sampling methods. Evolutionary Applications, 11, 1094–1119. 30026800 10.1111/eva.12600 PMC6050181

[jfb70361-bib-0016] Chapelle, V. , & Silvestre, F. (2022). Population epigenetics: The extent of DNA methylation variation in wild animal populations. Epigenomes, 6, 31. 36278677 10.3390/epigenomes6040031 PMC9589984

[jfb70361-bib-0017] Chen, S. , Li, N. , Safiul Azam, F. M. , Ao, L. , Li, N. , Wang, J. , Zou, Y. , Li, R. , & Prodhan, Z. H. (2025). Comparative transcriptome analysis of albino northern snakehead (*Channa argus*) reveals its various collagen‐related DEGs in caudal fin cells. PLoS One, 19, e0315996. 10.1371/journal.pone.0315996 PMC11687805 39739744

[jfb70361-bib-0018] Colombo, G. , & Grandi, G. (1996). Histological study of the development and sex differentiation of the gonad in the European eel. Journal of Fish Biology, 48, 493–512.

[jfb70361-bib-0019] Colombo, G. , Grandi, G. , & Rossi, R. (1984). Gonad differentiation and body growth in *Anguilla anguilla* L. Journal of Fish Biology, 24, 215–228.

[jfb70361-bib-0020] Conde, D. A. , Colchero, F. , Zarza, H. , Christensen, N. L. , Sexton, J. O. , Manterola, C. , Chávez, C. , Rivera, A. , Azuara, D. , & Ceballos, G. (2010). Sex matters: Modeling male and female habitat differences for jaguar conservation. Biological Conservation, 143, 1980–1988.

[jfb70361-bib-0021] Cooke, S. J. , Nguyen, V. M. , Murchie, K. J. , Thiem, J. D. , Donaldson, M. R. , Hinch, S. G. , Brown, R. S. , & Fisk, A. (2013). To tag or not to tag: Animal welfare, conservation, and stakeholder considerations in fish tracking studies that use electronic tags. Journal of International Wildlife Law & Policy, 16, 352–374.

[jfb70361-bib-0022] Crowley, P. H. , Labonne, J. , Bolliet, V. , Daverat, F. , & Bardonnet, A. (2022). Implications of stress‐mediated environmental sex determination for declining eel populations. Reviews in Fish Biology and Fisheries, 32, 1157–1186.

[jfb70361-bib-0023] da Silva, F. F. G. , Tveiten, H. , Maugars, G. , Lafont, A.‐G. , Dufour, S. , Støttrup, J. G. , Kjørsvik, E. , & Tomkiewicz, J. (2018). Differential expression of gonadotropin and estrogen receptors and oocyte cytology during follicular maturation associated with egg viability in European eel (*Anguilla anguilla*). Comparative Biochemistry and Physiology Part A: Molecular & Integrative Physiology, 221, 44–54. 10.1016/j.cbpa.2018.03.010 29597012

[jfb70361-bib-0024] Davey, A. J. H. , & Jellyman, D. J. (2005). Sex determination in freshwater eels and management options for manipulation of sex. Reviews in Fish Biology and Fisheries, 15, 37–52.

[jfb70361-bib-0025] Denis, J. , Mahé, K. , & Amara, R. (2022). Abundance and growth of the European eels (*Anguilla anguilla* Linnaeus, 1758) in small estuarine habitats from the eastern English Channel. Fishes, 7, 213.

[jfb70361-bib-0026] Dirks, R. P. , Burgerhout, E. , Brittijn, S. A. , de Wijze, D. L. , Ozupek, H. , Tuinhof‐Koelma, N. , Minegishi, Y. , Jong‐Raadsen, S. A. , Spaink, H. P. , & van den Thillart, G. E. E. J. M. (2014). Identification of molecular markers in pectoral fin to predict artificial maturation of female European eels (*Anguilla anguilla*). General and Comparative Endocrinology, 204, 267–276. 24992558 10.1016/j.ygcen.2014.06.023

[jfb70361-bib-0027] Drouineau, H. , Durif, C. , Castonguay, M. , Mateo, M. , Rochard, E. , Verreault, G. , Yokouchi, K. , & Lambert, P. (2018). Freshwater eels: A symbol of the effects of global change. Fish and Fisheries, 19, 903–930.

[jfb70361-bib-0028] du Bureau Colombier, S. , Jacobs, L. , Gesset, C. , Elie, P. , & Lambert, P. (2015). Ultrasonography as a non‐invasive tool for sex determination and maturation monitoring in silver eels. Fisheries Research, 164, 50–58.

[jfb70361-bib-0029] Durif, C. , Guibert, A. , & Elie, P. (2009). Morphological discrimination of the silvering stages of the European eel. In American fisheries society symposium (pp. 103–111). American Fisheries Society.

[jfb70361-bib-0030] Edmands, S. (2021). Sex ratios in a warming world: Thermal effects on sex‐biased survival, sex determination, and sex reversal. Journal of Heredity, 112, 155–164. 33585893 10.1093/jhered/esab006

[jfb70361-bib-0031] Ellegren, H. , & Parsch, J. (2007). The evolution of sex‐biased genes and sex‐biased gene expression. Nature Reviews Genetics, 8, 689–698. 10.1038/nrg2167 17680007

[jfb70361-bib-0032] Erikstad, K. E. , Sandvik, H. , Reiertsen, T. K. , Bustnes, J. O. , & Strøm, H. (2013). Persistent organic pollution in a high‐Arctic top predator: Sex‐dependent thresholds in adult survival. Proceedings of the Royal Society B: Biological Sciences, 280, 20131483. 10.1098/rspb.2013.1483 PMC3768305 23966640

[jfb70361-bib-0033] Froman, N. , Genain, M.‐A. , Stevens, G. M. W. , & Pearce, G. P. (2023). Use of underwater contactless ultrasonography to elucidate the internal anatomy and reproductive activity of manta and devil rays (family: Mobulidae). Journal of Fish Biology, 103, 305–323. 37158279 10.1111/jfb.15423

[jfb70361-bib-0034] Gade, M. , Comfort, N. , & Re, D. B. (2021). Sex‐specific neurotoxic effects of heavy metal pollutants: Epidemiological, experimental evidence and candidate mechanisms. Environmental Research, 201, 111558. 34224706 10.1016/j.envres.2021.111558 PMC8478794

[jfb70361-bib-0035] Gamer, M. , & Lemon, J. (2019). Irr: Various coefficients of interrater reliability and agreement. R Package Version 0.84.1.

[jfb70361-bib-0036] Gantchoff, M. , Conlee, L. , & Belant, J. (2019). Conservation implications of sex‐specific landscape suitability for a large generalist carnivore. Diversity and Distributions, 25, 1488–1496.

[jfb70361-bib-0037] Geffroy, B. , & Bardonnet, A. (2016). Sex differentiation and sex determination in eels: Consequences for management. Fish and Fisheries, 17, 375–398.

[jfb70361-bib-0038] Geffroy, B. , Guilbaud, F. , Amilhat, E. , Beaulaton, L. , Vignon, M. , Huchet, E. , Rives, J. , Bobe, J. , Fostier, A. , Guiguen, Y. , & Bardonnet, A. (2016). Sexually dimorphic gene expressions in eels: Useful markers for early sex assessment in a conservation context. Scientific Reports, 6, 34041. 27658729 10.1038/srep34041 PMC5034313

[jfb70361-bib-0039] Geffroy, B. , & Wedekind, C. (2020). Effects of global warming on sex ratios in fishes. Journal of Fish Biology, 97, 596–606. 32524610 10.1111/jfb.14429

[jfb70361-bib-0040] Ginsberg, J. R. , & Milner‐Gulland, E. J. (1994). Sex‐biased harvesting and population dynamics in ungulates: Implications for conservation and sustainable use. Conservation Biology, 8, 157–166.

[jfb70361-bib-0041] Giroux, A. , Ortega, Z. , Oliveira‐Santos, L. G. R. , Attias, N. , Bertassoni, A. , & Desbiez, A. L. J. (2021). Sexual, allometric and forest cover effects on giant anteaters' movement ecology. PLoS One, 16, e0253345. 34407068 10.1371/journal.pone.0253345 PMC8372905

[jfb70361-bib-0042] Gopikrishna, G. (2023). Chapter 4 ‐ application of molecular markers in aquaculture. In W. S. Lakra , M. Goswami , & V. L. Trudeau (Eds.), Frontiers in aquaculture biotechnology (pp. 47–53). Academic Press.

[jfb70361-bib-0043] Hala, E. , Kule, M. , Kamberi, E. , Bakiu, R. , & Simon, J. (2025). Age and growth of European eel, *Anguilla anguilla* (Actinopterygii, Anguilliformes, Anguillidae), in the Karavasta lagoon, Albania. Acta Ichthyologica Et Piscatoria, 55, 259–271.

[jfb70361-bib-0044] Hess, M. C. , Inoue, K. , Tsakiris, E. T. , Hart, M. , Morton, J. , Dudding, J. , Robertson, C. R. , & Randklev, C. R. (2018). Misidentification of sex for *Lampsilis teres*, yellow sandshell, and its implications for mussel conservation and wildlife management. PLoS One, 13, e0197107. 29768469 10.1371/journal.pone.0197107 PMC5955573

[jfb70361-bib-0045] Hildebrandt, T. , Pitra, C. , Sömmer, P. , & Pinkowski, M. (1995). Sex identification in birds of prey by ultrasonography. Journal of Zoo and Wildlife Medicine, 26, 367–376.

[jfb70361-bib-0046] Höhne, L. , Pohlmann, J.‐D. , & Freese, M. (2023). Minimally invasive collection of biometric data including maturation stage on European eel using photography. Marine and Coastal Fisheries, 15, e10239.

[jfb70361-bib-0047] Holleley, C. E. , Whiteley, S. L. , Devloo‐Delva, F. , Bachler, A. , Llinas, J. , & Georges, A. (2023). Molecular sex identification for applications in conservation, industry and veterinary medicine. In O. F. Berry , C. E. Holleley , & S. N. Jarman (Eds.), Applied environmental genomics (pp. 74–101). CRC Press.

[jfb70361-bib-0048] Horiuchi, M. , Hagihara, S. , Kume, M. , Chushi, D. , Hasegawa, Y. , Itakura, H. , Yamashita, Y. , Adachi, S. , & Ijiri, S. (2022). Morphological and molecular gonadal sex differentiation in the wild Japanese eel *Anguilla japonica* . Cells, 11, 1554. 35563858 10.3390/cells11091554 PMC9105286

[jfb70361-bib-0049] Houdelet, C. , Blondeau‐Bidet, E. , Estevez‐Villar, M. , Mialhe, X. , Hermet, S. , Ruelle, F. , Dutto, G. , Bajek, A. , Bobe, J. , & Geffroy, B. (2023). Circulating microRNAs indicative of sex and stress in the European seabass (*Dicentrarchus labrax*): Toward the identification of new biomarkers. Marine Biotechnology, 25, 749–762. 37581865 10.1007/s10126-023-10237-0

[jfb70361-bib-0050] Hsu, H. Y. , Chuang, C. H. , Lu, I. H. , Lin, C. Y. , & Han, Y. S. (2023). Identification of sexually dimorphic genes in pectoral fin as molecular markers for assessing the sex of Japanese silver sels (*Anguilla japonica*). Zoological Studies, 62, e2. 37124871 10.6620/ZS.2023.62-02 PMC10131072

[jfb70361-bib-0051] Hsu, L. M. , & Field, R. (2003). Interrater agreement measures: Comments on Kappan, Cohen's kappa, Scott's π, and Aickin's α. Understanding Statistics, 2, 205–219.

[jfb70361-bib-0052] Hwang, J.‐A. , Park, J. , Kim, J. E. , Lee, J.‐H. , & Kim, H. S. (2022). Estradiol‐17 β levels as a tool for sex determination in farmed *Anguilla japonica* . Biochemical and Biophysical Research Communications, 634, 108–113. 36242916 10.1016/j.bbrc.2022.10.020

[jfb70361-bib-0053] Iversen, M. H. , Økland, F. , Thorstad, E. B. , & Finstad, B. (2013). The efficacy of Aqui‐S vet. (iso‐eugenol) and metomidate as anaesthetics in European eel (*Anguilla anguilla* L.), and their effects on animal welfare and primary and secondary stress responses. Aquaculture Research, 44, 1307–1316.

[jfb70361-bib-0054] Jacoby, D. M. P. , Casselman, J. M. , Crook, V. , DeLucia, M.‐B. , Ahn, H. , Kaifu, K. , Kurwie, T. , Sasal, P. , Silfvergrip, A. M. C. , Smith, K. G. , Uchida, K. , Walker, A. M. , & Gollock, M. J. (2015). Synergistic patterns of threat and the challenges facing global anguillid eel conservation. Global Ecology and Conservation, 4, 321–333.

[jfb70361-bib-0055] Janes, D. E. , Elsey, R. M. , Langan, E. M. , Valenzuela, N. , & Edwards, S. V. (2013). Sex‐biased expression of sex‐differentiating genes FOXL2 and FGF9 in American alligators, *Alligator mississippiensis* . Sexual Development, 7, 253–260. 23689672 10.1159/000350787 PMC3798014

[jfb70361-bib-0056] Jung, E. M. , Dinkel, J. , Verloh, N. , Brandenstein, M. , Stroszczynski, C. , Jung, F. , & Rennert, J. (2021). Wireless point‐of‐care ultrasound: First experiences with a new generation handheld device. Clinical Hemorheology and Microcirculation, 79, 463–474. 34151848 10.3233/CH-211197 PMC8764604

[jfb70361-bib-0057] Kendall, N. W. , & Quinn, T. P. (2013). Size‐selective fishing affects sex ratios and the opportunity for sexual selection in Alaskan sockeye salmon *Oncorhynchus nerka* . Oikos, 122, 411–420.

[jfb70361-bib-0058] Kirk, H. , & Freeland, J. R. (2011). Applications and implications of neutral versus non‐neutral markers in molecular ecology. International Journal of Molecular Sciences, 12, 3966–3988. 21747718 10.3390/ijms12063966 PMC3131602

[jfb70361-bib-0059] Kitano, J. , Ansai, S. , Takehana, Y. , & Yamamoto, Y. (2024). Diversity and convergence of sex determination mechanisms in teleost fish. Annual Review of Animal Biosciences, 12, 233–259. 37863090 10.1146/annurev-animal-021122-113935

[jfb70361-bib-0060] Komarnicki, G. J. K. (2000). Tissue, sex and age specific accumulation of heavy metals (Zn, Cu, Pb, Cd) by populations of the mole (*Talpa europaea* L.) in a central urban area. Chemosphere, 41, 1593–1602. 11057686 10.1016/s0045-6535(00)00018-7

[jfb70361-bib-0061] Komrakova, M. , Knorr, C. , Brenig, B. , Hoerstgen‐Schwark, G. , & Holtz, W. (2018). Sex discrimination in rainbow trout (*Oncorhynchus mykiss*) using various sources of DNA and different genetic markers. Aquaculture, 497, 373–379.

[jfb70361-bib-0062] Korstian, J. M. , Hale, A. M. , Bennett, V. J. , & Williams, D. A. (2013). Advances in sex determination in bats and its utility in wind‐wildlife studies. Molecular Ecology Resources, 13, 776–780. 23647806 10.1111/1755-0998.12118

[jfb70361-bib-0063] Kucharczyk, D. , Żarski, D. , Krejszeff, S. , Nowosad, J. , Biłas, M. , Targonska, K. , & Palinska‐Zarska, K. (2016). Use of an ultrasound device to determine sex and to perform gonad biopsy in the European eel *Anguilla anguilla* . Brazilian Journal of Veterinary Research and Animal Science, 53, 199–206.

[jfb70361-bib-0064] Laopichienpong, N. , Tawichasri, P. , Chanhome, L. , Phatcharakullawarawat, R. , Singchat, W. , Kantachumpoo, A. , Muangmai, N. , Suntrarachun, S. , Matsubara, K. , Peyachoknagul, S. , & Srikulnath, K. (2017). A novel method of caenophidian snake sex identification using molecular markers based on two gametologous genes. Ecology and Evolution, 7, 4661–4669. 28690796 10.1002/ece3.3057 PMC5496543

[jfb70361-bib-0065] Lennox, R. J. , Økland, F. , Mitamura, H. , Cooke, S. J. , & Thorstad, E. B. (2018). European eel *Anguilla anguilla* compromise speed for safety in the early marine spawning migration. ICES Journal of Marine Science, 75, 1984–1991.

[jfb70361-bib-0066] Li, M. , Xu, H. , Xu, W. , Zhou, Q. , Xu, X. , Zhu, Y. , Zheng, W. , Li, W. , Pang, Z. , & Chen, S. (2020). Isolation of a male‐specific molecular marker and development of a genetic sex identification technique in spotted knifejaw (*Oplegnathus punctatus*). Marine Biotechnology, 22, 467–474. 32424478 10.1007/s10126-020-09966-3

[jfb70361-bib-0067] Literman, R. , Badenhorst, D. , & Valenzuela, N. (2014). qPCR‐based molecular sexing by copy number variation in rRNA genes and its utility for sex identification in soft‐shell turtles. Methods in Ecology and Evolution, 5, 872–880.

[jfb70361-bib-0068] Macaulay, G. , Warren‐Myers, F. , Barrett, L. T. , Oppedal, F. , Føre, M. , & Dempster, T. (2021). Tag use to monitor fish behaviour in aquaculture: A review of benefits, problems and solutions. Reviews in Aquaculture, 13, 1565–1582.

[jfb70361-bib-0069] Macrì, F. , Rapisarda, G. , De Stefano, C. , De Majo, M. , Bottari, T. , & Aiudi, G. (2014). Coelioscopic investigation in European eels (*Anguilla anguilla*). Journal of Exotic Pet Medicine, 23, 147–151.

[jfb70361-bib-0070] Madenjian, C. P. , Rediske, R. R. , Krabbenhoft, D. P. , Stapanian, M. A. , Chernyak, S. M. , & O'Keefe, J. P. (2016). Sex differences in contaminant concentrations of fish: A synthesis. Biology of Sex Differences, 7, 42. 27594982 10.1186/s13293-016-0090-x PMC5010710

[jfb70361-bib-0071] Maiorano, L. , Boitani, L. , Chiaverini, L. , & Ciucci, P. (2017). Uncertainties in the identification of potential dispersal corridors: The importance of behaviour, sex, and algorithm. Basic and Applied Ecology, 21, 66–75.

[jfb70361-bib-0072] Mattson, N. S. (1991). A new method to determine sex and gonad size in live fishes by using ultrasonography. Journal of Fish Biology, 39, 673–677.

[jfb70361-bib-0073] Mayne, B. , Mustin, W. , Baboolal, V. , Casella, F. , Ballorain, K. , Barret, M. , Vanderklift, M. A. , Tucker, A. D. , & Berry, O. (2023). Differential methylation between sex in adult green sea turtle skin biopsies. Frontiers in marine Science, 10, 1169808.

[jfb70361-bib-0074] McCleave, J. D. , & Arnold, G. P. (1999). Movements of yellow‐ and silver‐phase European eels (*Anguilla anguilla* L.) tracked in the western North Sea. ICES Journal of Marine Science, 56, 510–536.

[jfb70361-bib-0075] McCoy, J. A. , Hamlin, H. J. , Thayer, L. , Guillette, L. J. , & Parrott, B. B. (2016). The influence of thermal signals during embryonic development on intrasexual and sexually dimorphic gene expression and circulating steroid hormones in American alligator hatchlings (*Alligator mississippiensis*). General and Comparative Endocrinology, 238, 47–54. 27080549 10.1016/j.ygcen.2016.04.011

[jfb70361-bib-0076] McHugh, M. L. (2012). Interrater reliability: The kappa statistic. Biochemia Medica (Zagreb), 22, 276–282. PMC3900052 23092060

[jfb70361-bib-0077] Milla, S. , Depiereux, S. , & Kestemont, P. (2011). The effects of estrogenic and androgenic endocrine disruptors on the immune system of fish: A review. Ecotoxicology, 20, 305–319. 21210218 10.1007/s10646-010-0588-7

[jfb70361-bib-0078] Milošević, D. , Mrdak, D. , & Causevic, D. (2022). Length‐weight relationship and condition factor of silver stage of European eel, *Anguilla anguilla* (Linnaeus, 1758) from Lake Skadar (Montenegro). Studia Marina, 35, 5–11.

[jfb70361-bib-0079] Minegishi, Y. , Aoyama, J. , Inoue, J. G. , Miya, M. , Nishida, M. , & Tsukamoto, K. (2005). Molecular phylogeny and evolution of the freshwater eels genus Anguilla based on the whole mitochondrial genome sequences. Molecular Phylogenetics and Evolution, 34, 134–146. 15579387 10.1016/j.ympev.2004.09.003

[jfb70361-bib-0080] Moghim, M. , Vajhi, A. R. , Veshkini, A. , & Masoudifard, M. (2002). Determination of sex and maturity in *Acipenser stellatus* by using ultrasonography. Journal of Applied Ichthyology, 18, 325–328.

[jfb70361-bib-0081] Morini, M. , Pasquier, J. , Dirks, R. , van den Thillart, G. , Tomkiewicz, J. , Rousseau, K. , Dufour, S. , & Lafont, A.‐G. (2015). Duplicated leptin receptors in two species of eel bring new insights into the evolution of the leptin system in vertebrates. PLoS One, 10, e0126008. 25946034 10.1371/journal.pone.0126008 PMC4422726

[jfb70361-bib-0082] Müller, A. V. , McEvoy, F. J. , Tomkiewicz, J. , Politis, S. N. , & Amigo, J. M. (2016). Ultrasonographic predictors of response of European eels (*Anguilla anguilla*) to hormonal treatment for induction of ovarian development. American Journal of Veterinary Research, 77, 478–486. 27111015 10.2460/ajvr.77.5.478

[jfb70361-bib-0083] Nakamura, M. (2010). The mechanism of sex determination in vertebrates—Are sex steroids the key‐factor? Journal of Experimental Zoology Part A: Ecological Genetics and Physiology, 313A, 381–398. 10.1002/jez.616 20623803

[jfb70361-bib-0084] Naqvi, S. , Godfrey, A. K. , Hughes, J. F. , Goodheart, M. L. , Mitchell, R. N. , & Page, D. C. (2019). Conservation, acquisition, and functional impact of sex‐biased gene expression in mammals. Science, 365, eaaw7317. 31320509 10.1126/science.aaw7317 PMC6896219

[jfb70361-bib-0085] Newman, D. M. , Jones, P. L. , & Ingram, B. A. (2008). Sexing accuracy and indicators of maturation status in captive Murray cod *Maccullochella peelii peelii* using non‐invasive ultrasonic imagery. Aquaculture, 279, 113–119.

[jfb70361-bib-0086] Ogburn, M. B. (2019). The effects of sex‐biased fisheries on crustacean sex ratios and reproductive output. Invertebrate Reproduction & Development, 63, 200–207.

[jfb70361-bib-0087] Ospina‐Álvarez, N. , & Piferrer, F. (2008). Temperature‐dependent sex determination in fish revisited: Prevalence, a single sex ratio response pattern, and possible effects of climate change. PLoS One, 3, e2837. 18665231 10.1371/journal.pone.0002837 PMC2481392

[jfb70361-bib-0088] Palmer, D. H. , Rogers, T. F. , Dean, R. , & Wright, A. E. (2019). How to identify sex chromosomes and their turnover. Molecular Ecology, 28, 4709–4724. 31538682 10.1111/mec.15245 PMC6900093

[jfb70361-bib-0089] Pankhurst, N. W. (1982). Relation of visual changes to the onset of sexual maturation in the European eel *Anguilla anguilla* (L.). Journal of Fish Biology, 21, 127–140.

[jfb70361-bib-0090] Philipp, D. P. , Childers, W. F. , & Whitt, G. S. (1979). Evolution of patterns of differential gene expression: A comparison of the temporal and spatial patterns of lsozyme locus expression in two closely related fish species (northern largemouth bass, *Micropterus salmoidessalmoides*, and smallmouth bass, *Micropterus dolomieui*). Journal of Experimental Zoology, 210, 473–487.

[jfb70361-bib-0091] Pike, C. , Crook, V. , & Gollock, M. (2020). *Anguilla Anguilla*. In *the IUCN Red List of Threatened Species 2020*: e.T60344A152845178.

[jfb70361-bib-0092] Piper, A. T. , Rosewarne, P. J. , Pike, C. , & Wright, R. M. (2023). The eel ascending: The influence of lateral slope, climbing substrate and flow rate on eel pass performance. Fishes, 8, 612.

[jfb70361-bib-0093] Piper, A. T. , Rosewarne, P. J. , Wright, R. M. , & Kemp, P. S. (2020). Using ‘trap and transport’ to facilitate seaward migration of landlocked European eel (*Anguilla anguilla*) from lakes and reservoirs. Fisheries Research, 228, 105567.

[jfb70361-bib-0094] Purwaningrum, M. , Nugroho, H. A. , Asvan, M. , Karyanti, K. , Alviyanto, B. , Kusuma, R. , & Haryanto, A. (2019). Molecular techniques for sex identification of captive birds. Veterinary World, 12, 1506–1513. 31749589 10.14202/vetworld.2019.1506-1513 PMC6813601

[jfb70361-bib-0095] Quinn, A. E. , Radder, R. S. , Sarre, S. D. , Georges, A. , Ezaz, T. , & Shine, R. (2009). Isolation and development of a molecular sex marker for *Bassiana duperreyi*, a lizard with XX/XY sex chromosomes and temperature‐induced sex reversal. Molecular Genetics and Genomics, 281, 665–672. 19277717 10.1007/s00438-009-0437-7

[jfb70361-bib-0096] R Core Team . (2024). R: A language and environment for statistical computing. R Foundation for Statistical Computing.

[jfb70361-bib-0097] Reed, C. C. , & Iozzo, R. V. (2002). The role of decorin in collagen fibrillogenesis and skin homeostasis. Glycoconjugate Journal, 19, 249–255. 12975602 10.1023/A:1025383913444

[jfb70361-bib-0098] Righton, D. , Piper, A. , Aarestrup, K. , Amilhat, E. , Belpaire, C. , Casselman, J. , Castonguay, M. , Díaz, E. , Dörner, H. , Faliex, E. , Feunteun, E. , Fukuda, N. , Hanel, R. , Hanzen, C. , Jellyman, D. , Kaifu, K. , McCarthy, K. , Miller, M. J. , Pratt, T. , … Gollock, M. (2021). Important questions to progress science and sustainable management of anguillid eels. Fish and Fisheries, 22, 762–788.

[jfb70361-bib-0099] Rovatsos, M. , Vukić, J. , Lymberakis, P. , & Kratochvíl, L. (2015). Evolutionary stability of sex chromosomes in snakes. Proceedings of the Royal Society B: Biological Sciences, 282, 20151992. 10.1098/rspb.2015.1992 PMC4707751 26702042

[jfb70361-bib-0100] Saaristo, M. , Tomkins, P. , Allinson, M. , Allinson, G. , & Wong, B. B. M. (2013). An androgenic agricultural contaminant impairs female reproductive behaviour in a freshwater fish. PLoS One, 8, e62782. 23671634 10.1371/journal.pone.0062782 PMC3643955

[jfb70361-bib-0101] Scarsella, G. E. , Gresham, J. D. , & Earley, R. L. (2018). Relationships between external sexually dimorphic characteristics and internal gonadal morphology in a sex‐changing fish. Journal of Zoology, 305, 133–140.

[jfb70361-bib-0102] Schuett, W. , Tregenza, T. , & Dall, S. R. X. (2010). Sexual selection and animal personality. Biological Reviews, 85, 217–246. 19922534 10.1111/j.1469-185X.2009.00101.x

[jfb70361-bib-0103] Sharba, S. , Sundh, H. , Sundell, K. , Benktander, J. , Santos, L. , Birchenough, G. , & Lindén, S. K. (2022). Rainbow trout gastrointestinal mucus, mucin production, mucin glycosylation and response to lipopolysaccharide. Fish & Shellfish Immunology, 122, 181–190. 35077869 10.1016/j.fsi.2022.01.031

[jfb70361-bib-0104] Shen, Z.‐G. , & Wang, H.‐P. (2018). Environmental sex determination and sex differentiation in teleosts – How sex is established. In H.‐P. Wang , F. Piferrer , & S.‐L. Chen (Eds.), Sex control in aquaculture (pp. 85–115). John Wiley & Sons.

[jfb70361-bib-0105] Simon, J. (2007). Age, growth, and condition of European eel (*Anguilla anguilla*) from six lakes in the river Havel system (Germany). ICES Journal of Marine Science, 64, 1414–1422.

[jfb70361-bib-0106] Smith, G. P. , Bronstein, J. L. , & Papaj, D. R. (2019). Sex differences in pollinator behavior: Patterns across species and consequences for the mutualism. Journal of Animal Ecology, 88, 971–985. 30921474 10.1111/1365-2656.12988

[jfb70361-bib-0107] Sönmez, B. , Bağda, E. , Candan, O. , & Yİlmaz, H. E. (2019). Sex determination in green turtle hatchlings: Geometric morphometry and molecular sex markers. Natural and Engineering Sciences, 4, 42–54.

[jfb70361-bib-0108] Soulsbury, C. D. , Gray, H. E. , Smith, L. M. , Braithwaite, V. , Cotter, S. C. , Elwood, R. W. , Wilkinson, A. , & Collins, L. M. (2020). The welfare and ethics of research involving wild animals: A primer. Methods in Ecology and Evolution, 11, 1164–1181.

[jfb70361-bib-0109] Sun, C. , Huang, J. , Wang, Y. , Zhao, X. , Su, L. , Thomas, G. W. C. , Zhao, M. , Zhang, X. , Jungreis, I. , Kellis, M. , Vicario, S. , Sharakhov, I. V. , Bondarenko, S. M. , Hasselmann, M. , Kim, C. N. , Paten, B. , Penso‐Dolfin, L. , Wang, L. , Chang, Y. , … Mueller, R. L. (2020). Genus‐wide characterization of bumblebee genomes provides insights into their evolution and variation in ecological and behavioral traits. Molecular Biology and Evolution, 38, 486–501. 10.1093/molbev/msaa240 PMC7826183 32946576

[jfb70361-bib-0110] Tarka, M. , Guenther, A. , Niemelä, P. T. , Nakagawa, S. , & Noble, D. W. A. (2018). Sex differences in life history, behavior, and physiology along a slow‐fast continuum: A meta‐analysis. Behavioral Ecology and Sociobiology, 72, 132. 30100667 10.1007/s00265-018-2534-2 PMC6060830

[jfb70361-bib-0111] Tesch, F.‐W. (2003). Post‐larval ecology and behaviour. In The eel (pp. 119–212). Blackwell Publishing.

[jfb70361-bib-0112] Trancart, T. , Feunteun, E. , Danet, V. , Carpentier, A. , Mazel, V. , Charrier, F. , Druet, M. , & Acou, A. (2018). Migration behaviour and escapement of European silver eels from a large lake and wetland system subject to water level management (grand‐lieu lake, France): New insights from regulated acoustic telemetry data. Ecology of Freshwater Fish, 27, 570–579.

[jfb70361-bib-0113] Turcu, M.‐C. , Paștiu, A. I. , Bel, L.‐V. , & Pusta, D. L. (2023). Minimally invasive sampling methods for molecular sexing of wild and companion birds. Animals, 13, 3417. 37958172 10.3390/ani13213417 PMC10648277

[jfb70361-bib-0114] Uusi‐Heikkilä, S. (2020). Implications of size‐selective fisheries on sexual selection. Evolutionary Applications, 13, 1487–1500. 32684971 10.1111/eva.12988 PMC7359828

[jfb70361-bib-0115] van Ginneken, V. , & Niemantsverdriet, P. (2017). Is global warming the cause for the dwindling European eel population. Oceanography & Fisheries, 2, 55559710.

[jfb70361-bib-0116] Walsh, C. T. , & Pease, B. C. (2002). The use of clove oil as an anaesthetic for the longfinned eel, *Anguilla reinhardtii* (Steindachner). Aquaculture Research, 33, 627–635.

[jfb70361-bib-0117] Wearmouth, V. J. , & Sims, D. W. (2008). Sexual segregation in marine fish, reptiles, birds and mammals: Behaviour patterns, mechanisms and conservation implications. In Advances in marine biology (pp. 107–170). Academic Press. 10.1016/S0065-2881(08)00002-3 18929064

[jfb70361-bib-0118] Whitehead, A. , & Crawford, D. L. (2006). Variation within and among species in gene expression: Raw material for evolution. Molecular Ecology, 15, 1197–1211. 16626448 10.1111/j.1365-294X.2006.02868.x

[jfb70361-bib-0119] Whittamore, J. M. , Bloomer, C. , Hanna, G. M. , & McCarthy, I. D. (2010). Evaluating ultrasonography as a non‐lethal method for the assessment of maturity in oviparous elasmobranchs. Marine Biology, 157, 2613–2624.

[jfb70361-bib-0120] Willemse, J. J. (1979). Guide to the internal morphology of the European eel, *Anguilla anguilla* (L.) (Pisces, Teleostei). Aquaculture, 17, 91–103.

[jfb70361-bib-0121] Wilson, C. A. , Priyanka , Titus, T. , Batzel, P. , Postlethwait, J. H. , & Raman, R. (2019). A search for sex‐linked loci in the agamid lizard, *Calotes versicolor* . Sexual Development, 13, 143–150. 31247625 10.1159/000500465

[jfb70361-bib-0122] Yeh, L.‐K. , Liu, C.‐Y. , Chien, C.‐L. , Converse, R. L. , Kao, W. W. Y. , Chen, M.‐S. , Hu, F.‐R. , Hsieh, F.‐J. , & Wang, I. J. (2008). Molecular analysis and characterization of zebrafish keratocan (zKera) gene. Journal of Biological Chemistry, 283, 506–517. 17965408 10.1074/jbc.M707656200

[jfb70361-bib-0123] Yu, M. , Xie, Q.‐P. , Wei, F.‐L. , Wu, X.‐F. , Xu, W.‐T. , Zhan, W. , Liu, F. , Guo, D.‐D. , Niu, B.‐L. , & Lou, B. (2022). Development and identification of a sex‐specific molecular marker in Dai‐qu stock large yellow croaker (*Larimichthys crocea*). Aquaculture, 555, 738172.

[jfb70361-bib-0124] Zhang, B. , Zhao, N. , Jia, L. , Peng, K. , Che, J. , Li, K. , He, X. , Sun, J. , & Bao, B. (2019). Seminal plasma exosomes: Promising biomarkers for identification of male and pseudo‐males in *Cynoglossus semilaevis* . Marine Biotechnology, 21, 310–319. 30863906 10.1007/s10126-019-09881-2

[jfb70361-bib-0125] Zhou, L. , Liu, F. , Chen, J. , Yang, R. , Li, J. , Wang, Z. , & Cai, M. (2023). Comparative transcriptome analysis reveals sex bias in expression patterns of genes related to sex steroids and immunity in the skin of spinyhead croaker *Collichthys lucidus* . Journal of Fish Biology, 103, 4–12. 37054975 10.1111/jfb.15405

