# Supplementary material for: Assessment of two minimally invasive methodologies for sex identification in the European eel, Anguilla anguilla
Source: J Fish Biol. 2026 Feb 12;108(6):1943–59. doi: 10.1111/jfb.70361 (PMC13357247; doi:10.1111/jfb.70361)
Supplement: Supplementary file 1 — TABLE S1. Ultrasound systems used for sex identification in the European eel, Anguilla anguilla. TABLE S2. CT values between undiluted and diluted samples for arp HK and dcn genes. TABLE S3. Eel ID and histological sex identification with 2ΔΔC T values for dcn, LOC111853410 and kera genes for a subsample of 14 eels. TABLE S4. CT values for arp, LOC111853410, kera and dcn genes for the initial exploration of expression values across 13 animals. [file JFB-108-1943-s002.docx]

Assessment of two minimally invasive methodologies for sex identification in European eel, *Anguilla anguilla.*

Michael J. Williamson^1,2,3^, Jack A. Brand^1,4^, Kevin Hopkins^1^, Luke O’Connor^5^, Matthew W. Perkins^1^, Christopher Sergeant^1^, Simon Spiro^5^, Taina Strike^5^, Jessica Whinfield^6,7^, Rosie S. Williams^1,2,3^, Ethan Wrigglesworth^5^, Rosalind M. Wright^8^ & Adam T. Piper^1,2,9^

^1^Institute of Zoology, Zoological Society of London, Regent’s Park, NW1 4RY, London, UK

^2^Department of Genetics, Evolution and Environment, University College London, Darwin Building, 99-105 Gower Street, London WC1E 6BT, UK

^3^Centre for Ecology and Conservation, College of Life and Environmental Sciences, University of Exeter, Penryn Campus, Penryn, Cornwall TR10 9FE, UK

^4^Department of Wildlife, Fish, and Environmental Studies, Swedish University of Agricultural Sciences, Umeå, 907 36, Sweden

^5^Zoological Society of London, Regent’s Park, London, NW1 4RY, UK

^6^The Harry Butler Institute, Murdoch University, Murdoch, Western Australia, Australia

^7^Taronga Conservation Society Australia, Mosman, New South Wales, Australia

^8^Environment Agency, Rivers House, Threshelfords Business Park, Inworth Road, Feering, CO5 9SE, UK

^9^Scottish Centre for Ecology and the Natural Environment, School of Biodiversity, One Health and Veterinary Medicine, University of Glasgow, Glasgow G63 0AW, UK

This supplementary material contains:

Table S1 Ultrasound systems used for sex identification in European eel, *Anguilla anguilla.*

Table S2 CT values between undiluted and diluted samples for *arp* HK and *dcn* genes

Table S3 Eel ID and histological sex identification with 2^ΔΔC^_T_ values for dcn, LOC111853410 and kera genes for a subsample of 14 eels.

Table S4 CT values for *arp*, LOC111853410, *kera*, and *dcn* genes for the initial exploration of expression values across 13 animals.

**Table S1** Ultrasound systems used for sex identification in European eel, *Anguilla anguilla.*

| **Ultrasound machine** | GE Versana Active portable ultrasound (General Electric Medical Systems) | GE VScan Air (General Electric Medical Systems) wireless dual-headed portable transducer |
| --- | --- | --- |
| **Transducer** | GE L8-18i-RS | Linear 3-12 MHz |
| **Device paired to** | N/A | 11-inch Samsung Galaxy tablet S9 |
| **Frequency used** | 16 MHz | Vascular Scan setting |
| **Depth used** | 0.5 - 3cm | 0.5 - 3cm |
| **Gain used** | 30% and 40% | Vascular Scan setting |
| **Cost** | ~ £21,000 | ~ £4,900 |

**Table S2** CT values between undiluted and diluted samples for *arp* and *dcn* genes. Sample IDs with the suffix “1:10” indicate diluted samples at a ratio of 1:10.

|  | *arp* | *dcn* |
| --- | --- | --- |
| XT721/23 | 21.459457 | 27.934507 |
| XT721/23_1:10 | 23.973274 | 31.446922 |
| XT722/23 | 21.475756 | 32.575108 |
| XT722/23_1:10 | 26.148993 | undetermined |

**Table S3** Eel ID and histological sex identification with 2^ΔΔC^_T_ values for dcn, LOC111853410 and kera genes for a subsample of 14 eels.

| Eel_ID | Histology | dcn | LOC111853410 | kera |
| --- | --- | --- | --- | --- |
| XT721/23 | F | female reference | | |
| XT722/23 | F | -2.624 | 1.923 | 3.372 |
| XT723/23 | M | 0.953 | 1.608 | -0.723 |
| XT724/23 | M | 2.195 | 4.977 | failed |
| XT725/23 | F | 1.169 | 0.711 | 3.419 |
| XT726/23 | M | 4.74 | 3.326 | 1.706 |
| XT727/23 | F | 2.804 | -0.333 | 1.16 |
| XT728/23 | M | 2.55 | 3.054 | 1.745 |
| XT729/23 | M | 2.962 | 0.614 | 5.303 |
| XT730/23 | F | 2.533 | -4.337 | -0.553 |
| XT731/23 | NO GONAD | 5.342 | 0.174 | 3.477 |
| XT732/23 | F | 0.625 | 2.216 | 1.105 |
| XT733/23 | NO GONAD | 2.276 | 4.184 | 4.706 |
| XT734/23 | F | 1.991 | 2.291 | 0.894 |

**Table S4** CT values for *arp*, LOC111853410, *kera*, and *dcn* genes for the initial exploration of expression values across 13 animals.

| Sample ID | *arp* | LOC111853410 | *kera* | *dcn* |
| --- | --- | --- | --- | --- |
| XT721/23 | 19.49 | 31.28 | 33.19 | 29.74 |
| XT722/23 | 24.08 | 35.94 | 36.40 | 33.60 |
| XT723/23 | 17.18 | 29.37 | 33.61 | 27.39 |
| XT724/23 | 21.18 | 29.99 | Undetermined | 30.57 |
| XT725/23 | 20.04 | 33.12 | 32.32 | 30.76 |
| XT726/23 | 22.97 | 33.43 | 36.96 | 32.07 |
| XT727/23 | 19.95 | 34.07 | 34.49 | 29.07 |
| XT728/23 | 18.69 | 29.43 | 32.64 | 27.69 |
| XT729/23 | 18.06 | 31.24 | 28.46 | 25.43 |
| XT730/23 | 17.66 | 35.79 | 33.91 | 24.72 |
| XT731/23 | 19.46 | 33.07 | 31.68 | 25.09 |
| XT732/23 | 17.31 | 28.89 | 31.91 | 26.78 |
| XT733/23 | 21.79 | 31.39 | 32.78 | 29.34 |
| XT734/23 | 17.49 | 28.99 | 32.30 | 25.62 |
