# Supplementary material for: Assessment of two minimally invasive methodologies for sex identification in the European eel, Anguilla anguilla
Source: J Fish Biol. 2026 Feb 12;108(6):1943–59. doi: 10.1111/jfb.70361 (PMC13357247; doi:10.1111/jfb.70361)
Supplement: Supplementary file 2 — DATA S1. Supporting Information. [file JFB-108-1943-s003.pdf]

Supporting Information 2 – Female Images

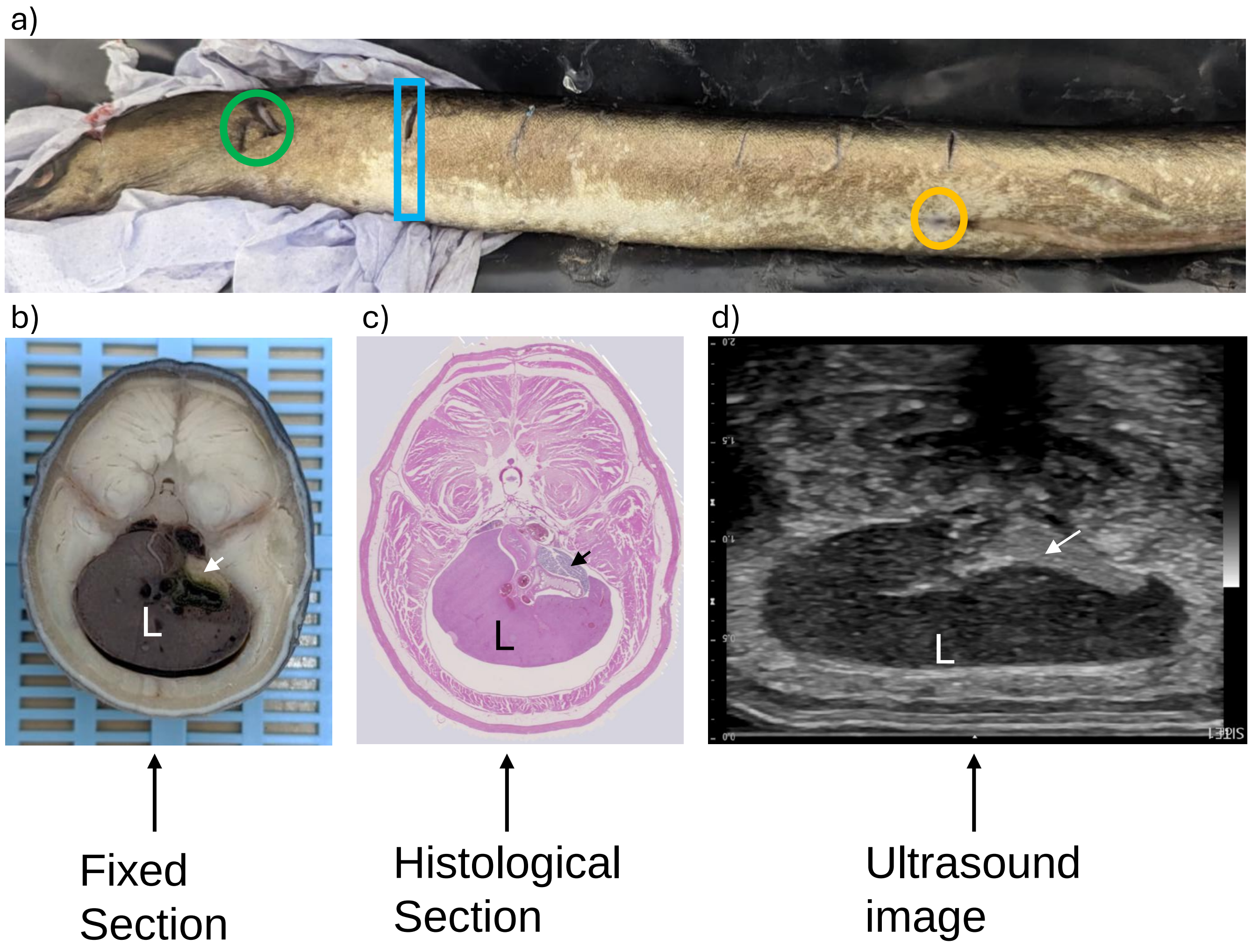

Key

– Pec fin / external gill slit

– Scanning site

– Anal pore

– Black / White Arrow - Indicator of presence and location of gonad

F – Fat
GIT – Gastrointestinal Tract
GB – Gall Bladder
L – Liver
K – Kidneys
S – Spleen
SB – Bladder
V – Vent / Anal Pore

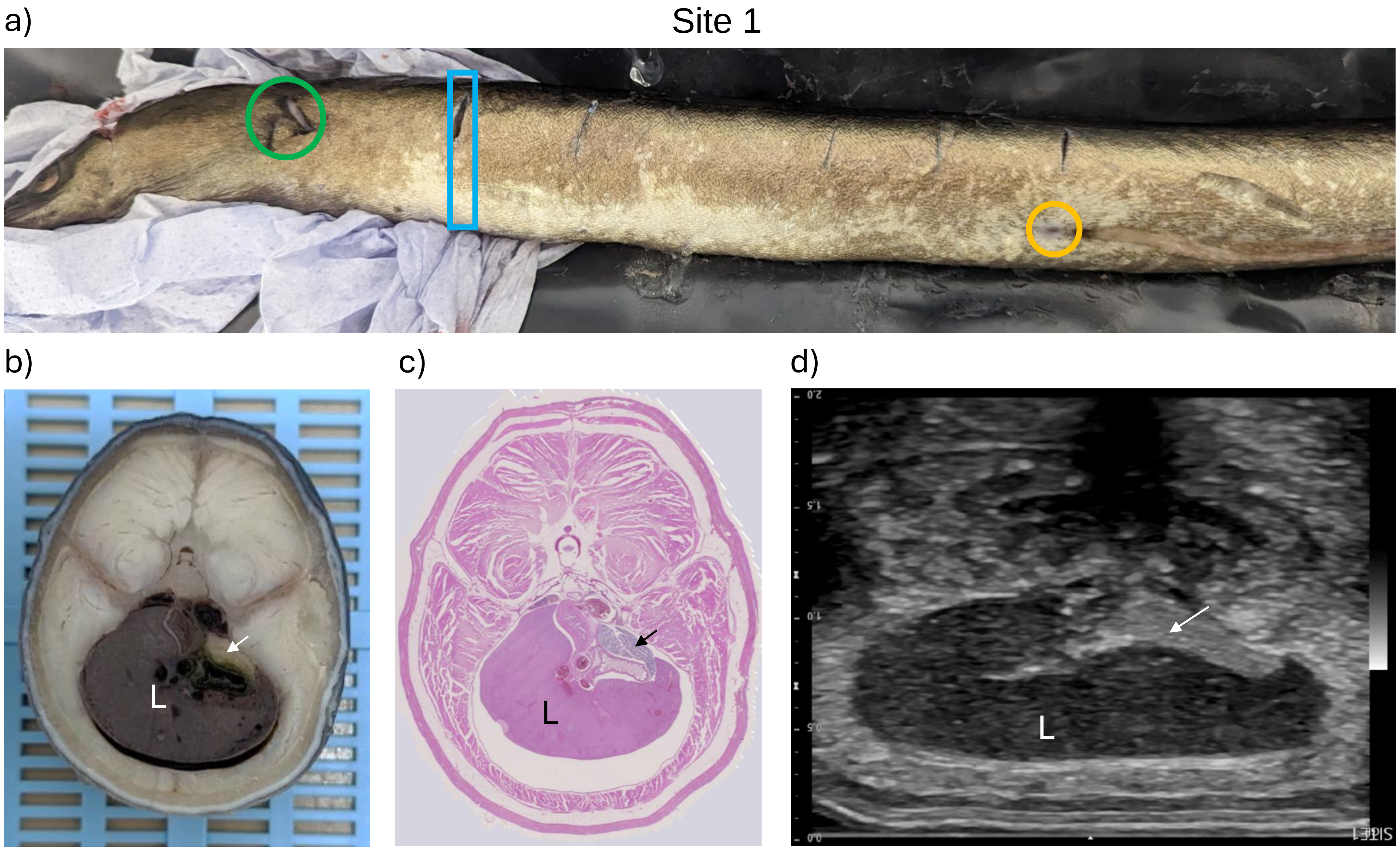

Figure S1. a) Image of female eel and location of scan at site 1. Scanning site is highlighted in a blue rectangle, pectoral fin and gill vent located in the green circle and anal pore located in yellow circle. Fixed section (b), histological section (c) and ultrasound image (d) at that site shown. Location of liver (L) indicated on all three images, with presence of gonad indicated by white or black arrow.

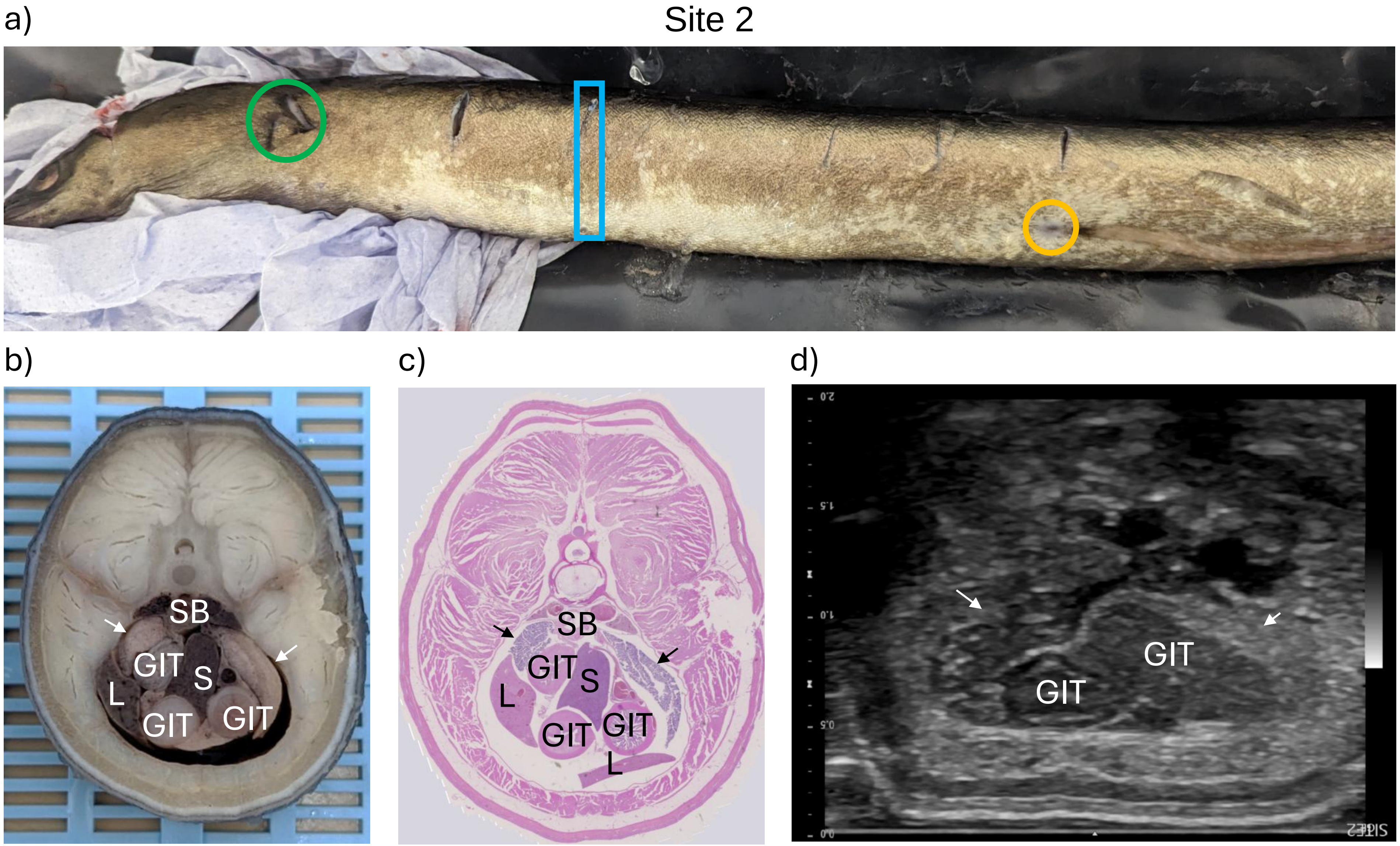

Figure S2. a) Image of female eel and location of scan at site 2. Scanning site is highlighted in a blue rectangle, pectoral fin and gill vent located in the green circle and anal pore located in yellow circle. Fixed section (b), histological section (c) and ultrasound image (d) at that site shown. Location of gastrointestinal tract (GIT), liver (L), spleen (S) and swim bladder (SB) indicated on all three images, with presence of gonad indicated by white or black arrow.

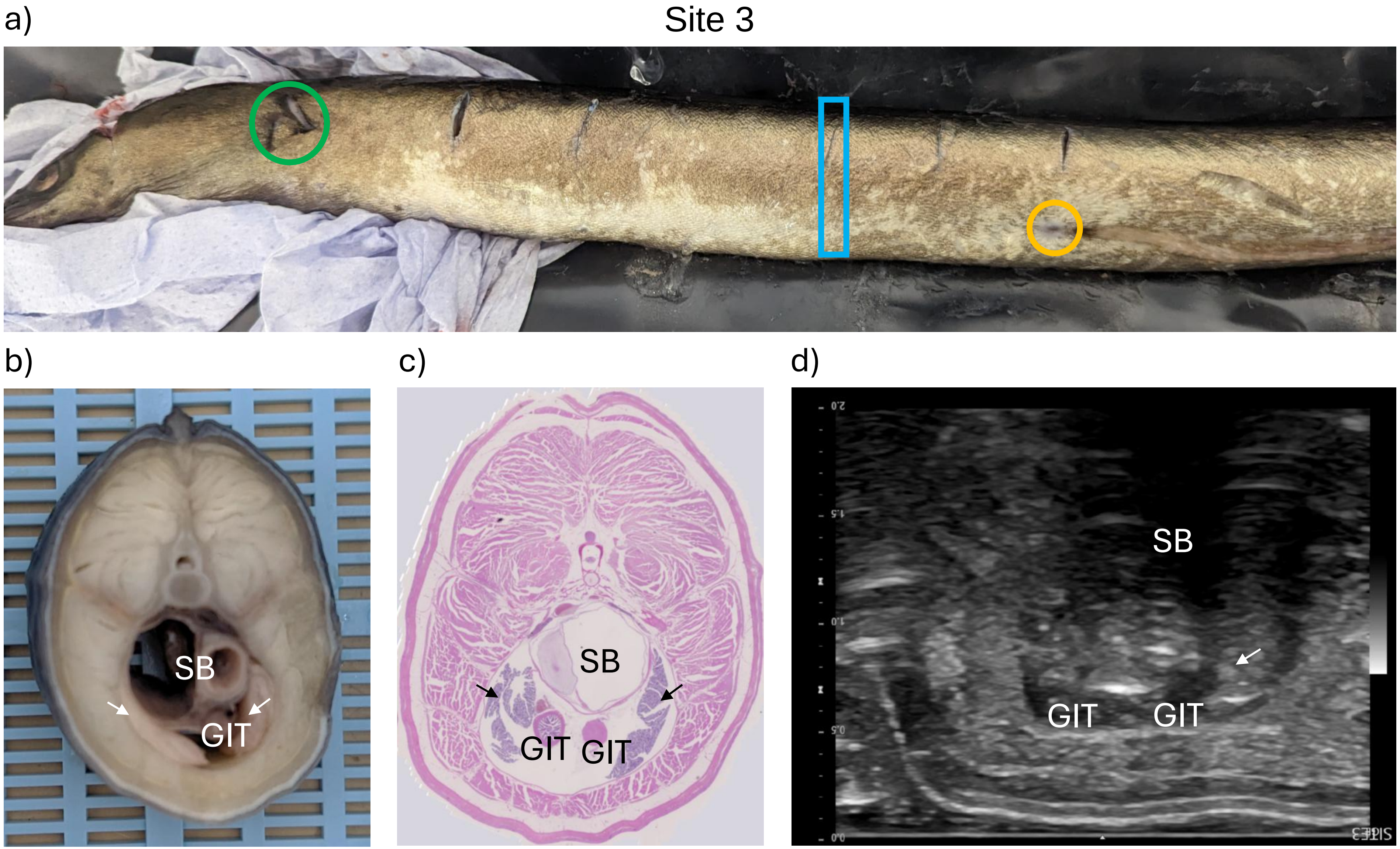

Figure S3. a) Image of female eel and location of scan at site 3. Scanning site is highlighted in a blue rectangle, pectoral fin and gill vent located in the green circle and anal pore located in yellow circle. Fixed section (b), histological section (c) and ultrasound image (d) at that site shown. Location of gastrointestinal tract (GIT), and swim bladder (SB) indicated on all three images, with presence of gonad indicated by white or black arrow.

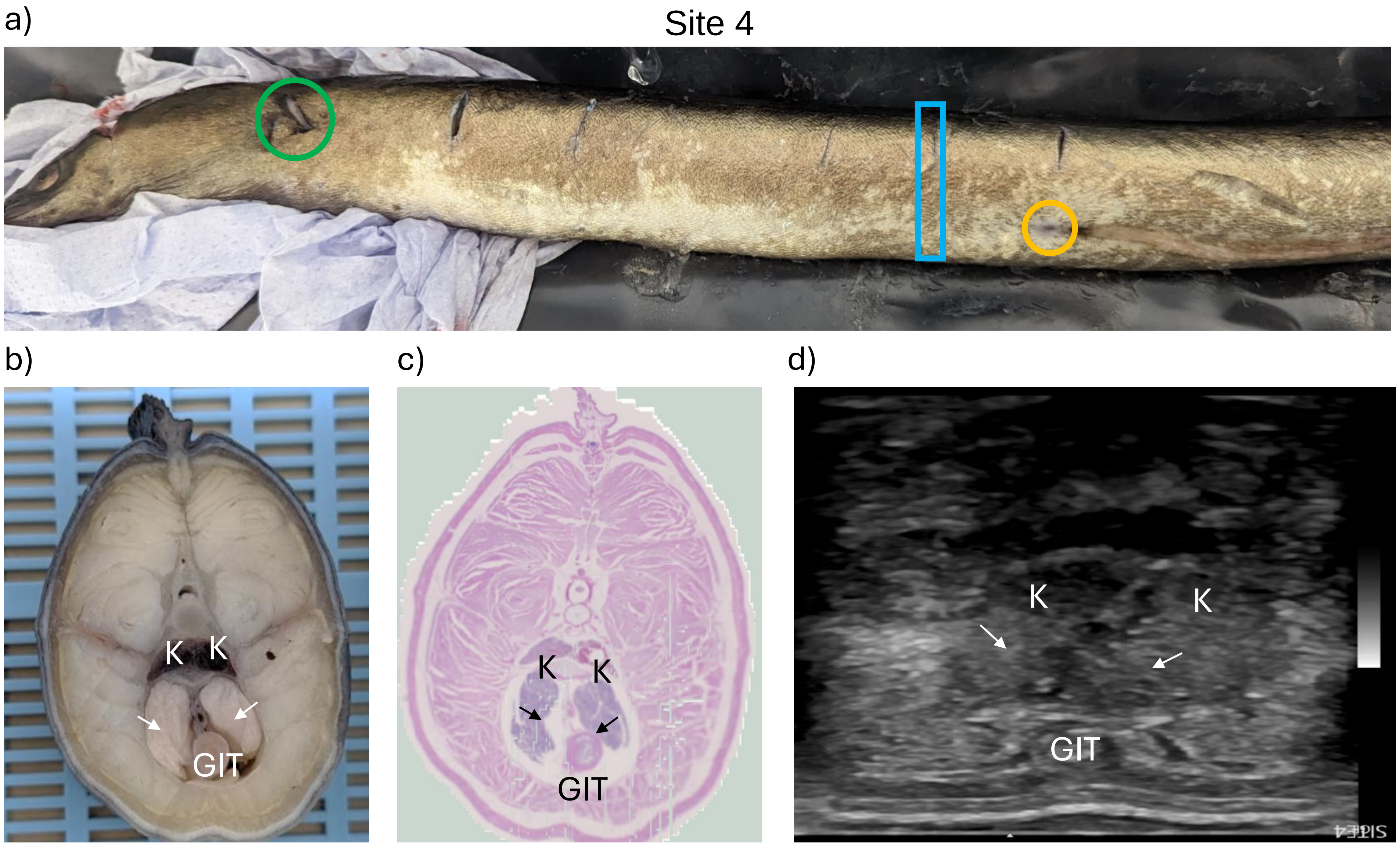

Figure S4. a) Image of female eel and location of scan at site 4. Scanning site is highlighted in a blue circle, pectoral fin and gill vent located in the green circle and anal pore located in yellow circle. Fixed section (b), histological section (c) and ultrasound image (d) at that site shown. Location of gastrointestinal tract (GIT), and kidneys (K) indicated on all three images, with presence of gonad indicated by white or black arrow.

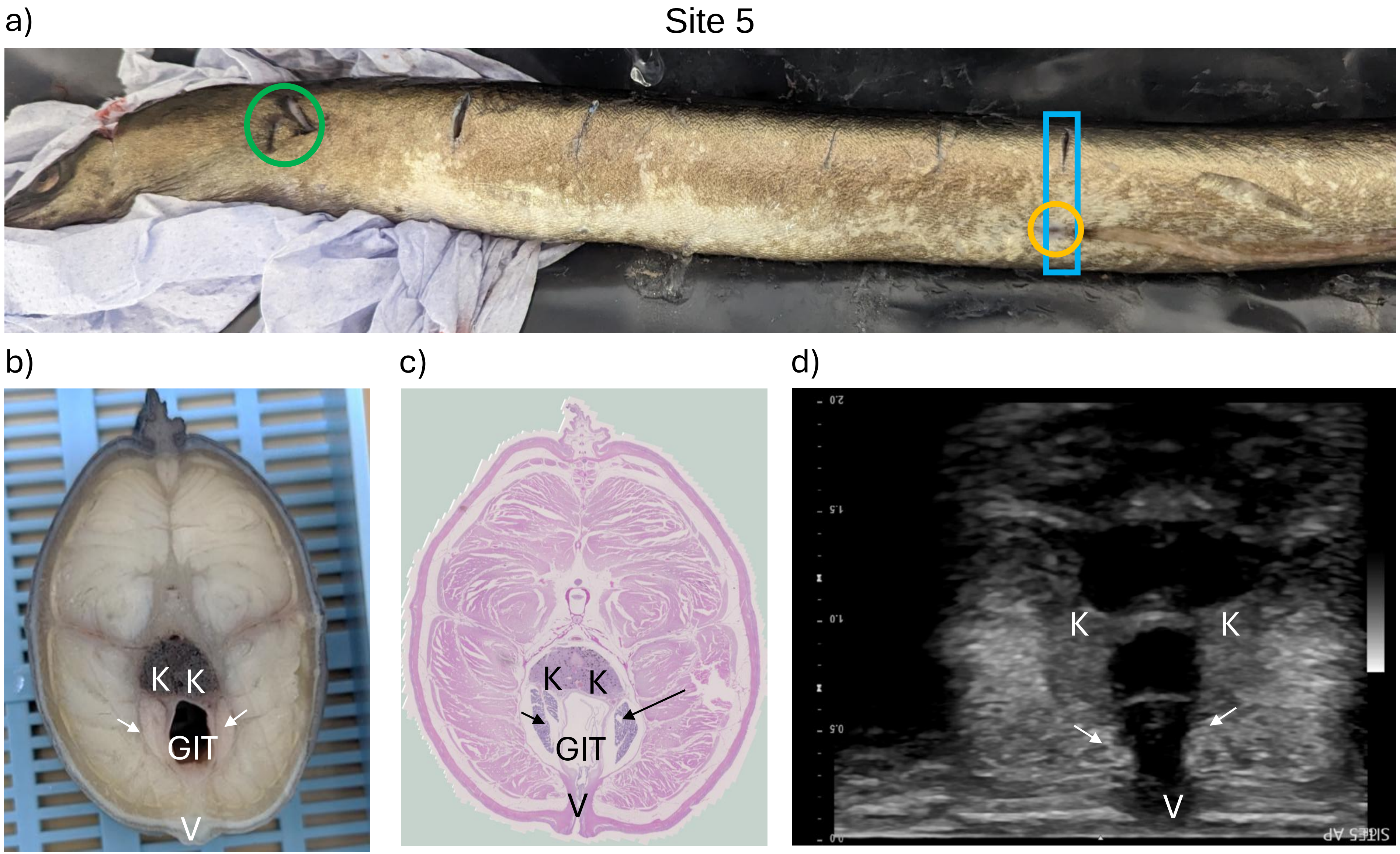

Figure S5. a) Image of female eel and location of scan at site 5. Scanning site is highlighted in a blue circle, pectoral fin and gill vent located in the green circle and anal pore located in yellow circle. Fixed section (b), histological section (c) and ultrasound image (d) at that site shown. Location of gastrointestinal tract (GIT), kidneys (K) and anal pore/vent (V) indicated on all three images, with presence of gonad indicated by white or black arrow.
