# Supplementary material for: Assessment of two minimally invasive methodologies for sex identification in the European eel, Anguilla anguilla
Source: J Fish Biol. 2026 Feb 12;108(6):1943–59. doi: 10.1111/jfb.70361 (PMC13357247; doi:10.1111/jfb.70361)
Supplement: Supplementary file 3 — DATA S2. Supporting Information. [file JFB-108-1943-s001.pdf]

Supporting Information 3 – Male Images

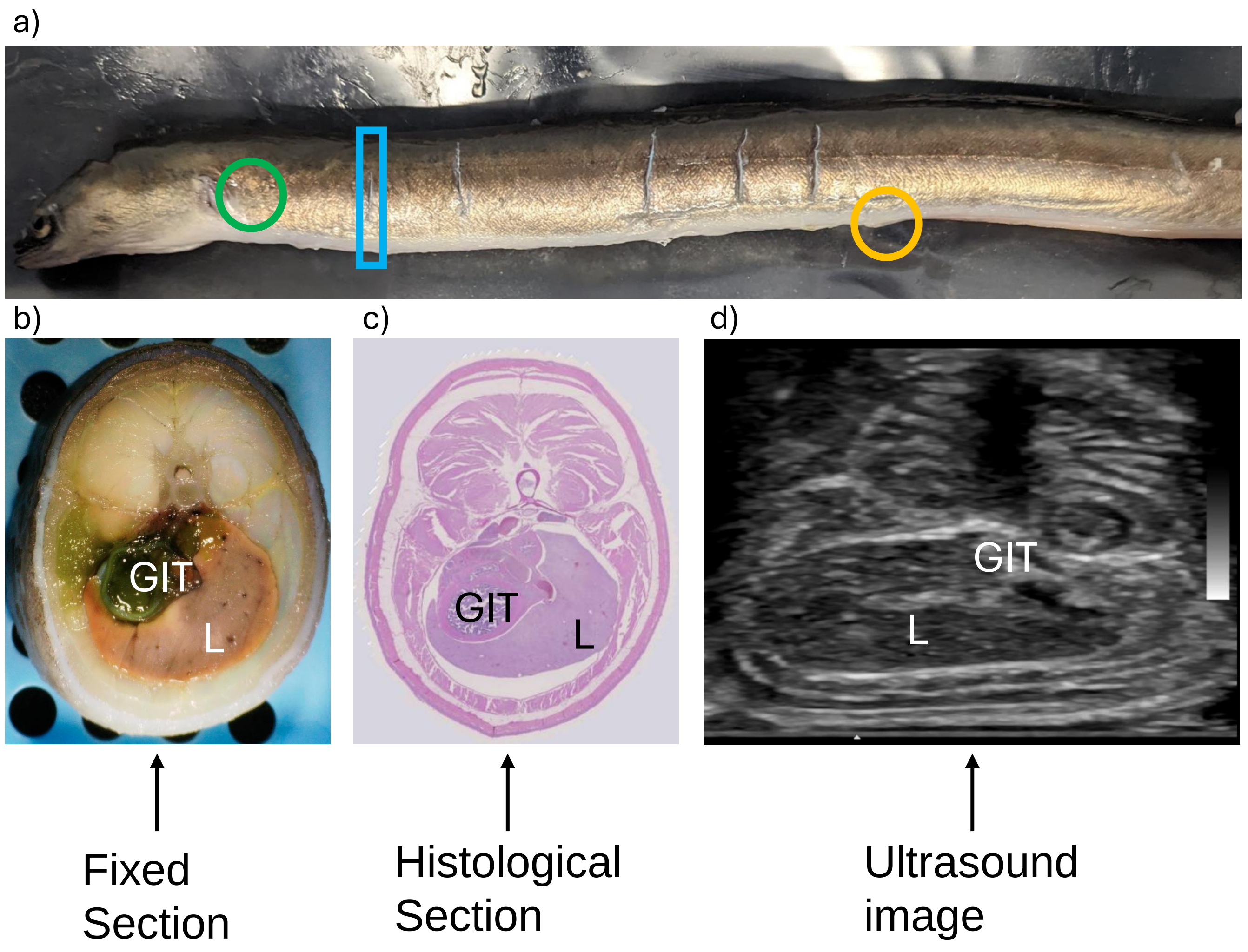

Key

– Pec fin/ external gill slit

– Scanning site

– Anal pore

– Black / White Arrow - Indicator of presence and location of gonad

F – Fat
GIT – Gut
GB – Gall Bladder
L – Liver
K – Kidneys
S – Spleen
SB – Bladder
V – Vent / Anal Pore

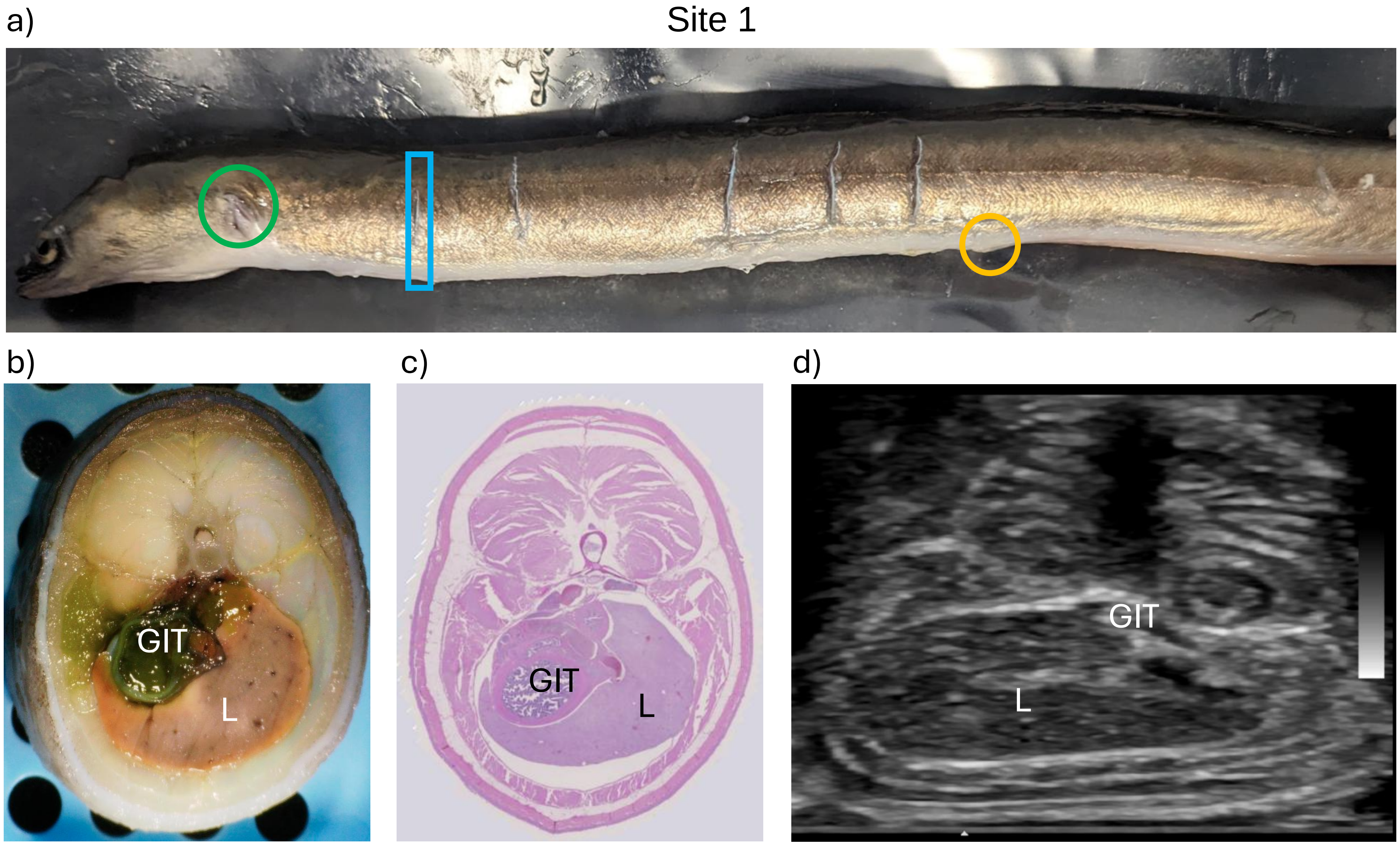

Figure S1. a) Image of male eel and location of scan at Site 1. Scanning site is highlighted in a blue rectangle, pectoral fin and gill vent highlighted in the green circle and anal pore located in yellow circle. Fixed section (b), histological section (c) and ultrasound image (d) at that site shown. Location of gastrointestinal tract (GIT) and liver (L) indicated on all three images. No gonad visible in any image, indicating that the individual is male.

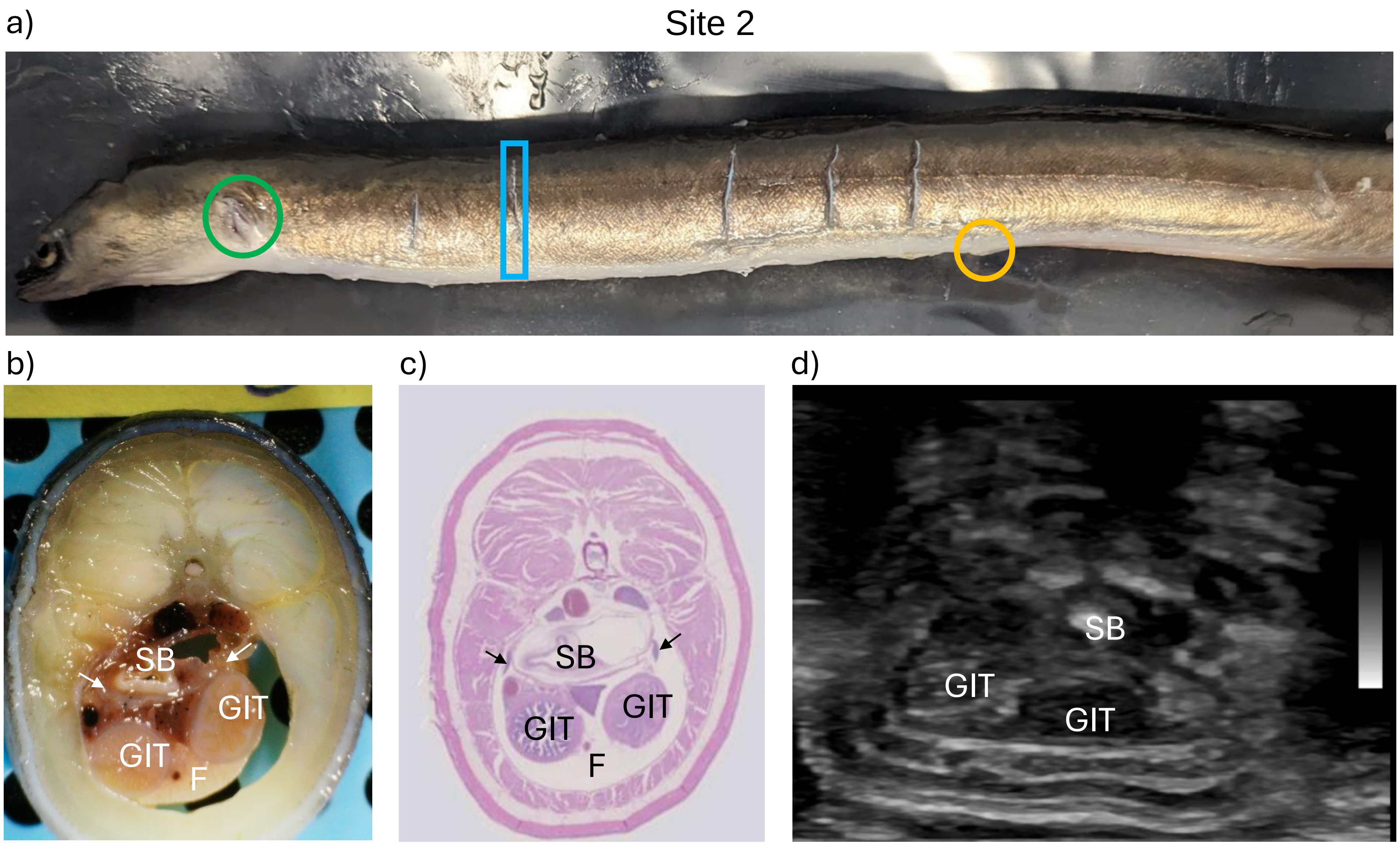

Figure S2. a) Image of male eel and location of scan at Site 2. Scanning site is highlighted in a blue rectangle, pectoral fin and gill vent highlighted in the green circle and anal pore located in yellow circle. Fixed section (b), histological section (c) and ultrasound image (d) at that site shown. Location of fat (F), gastrointestinal tract (GIT) and swim bladder (SB) indicated on all three images with presence of gonad indicated by white or black arrow. Small gonad size from fixed and histological sections and no gonad visible in ultrasound indicating that the individual is male.

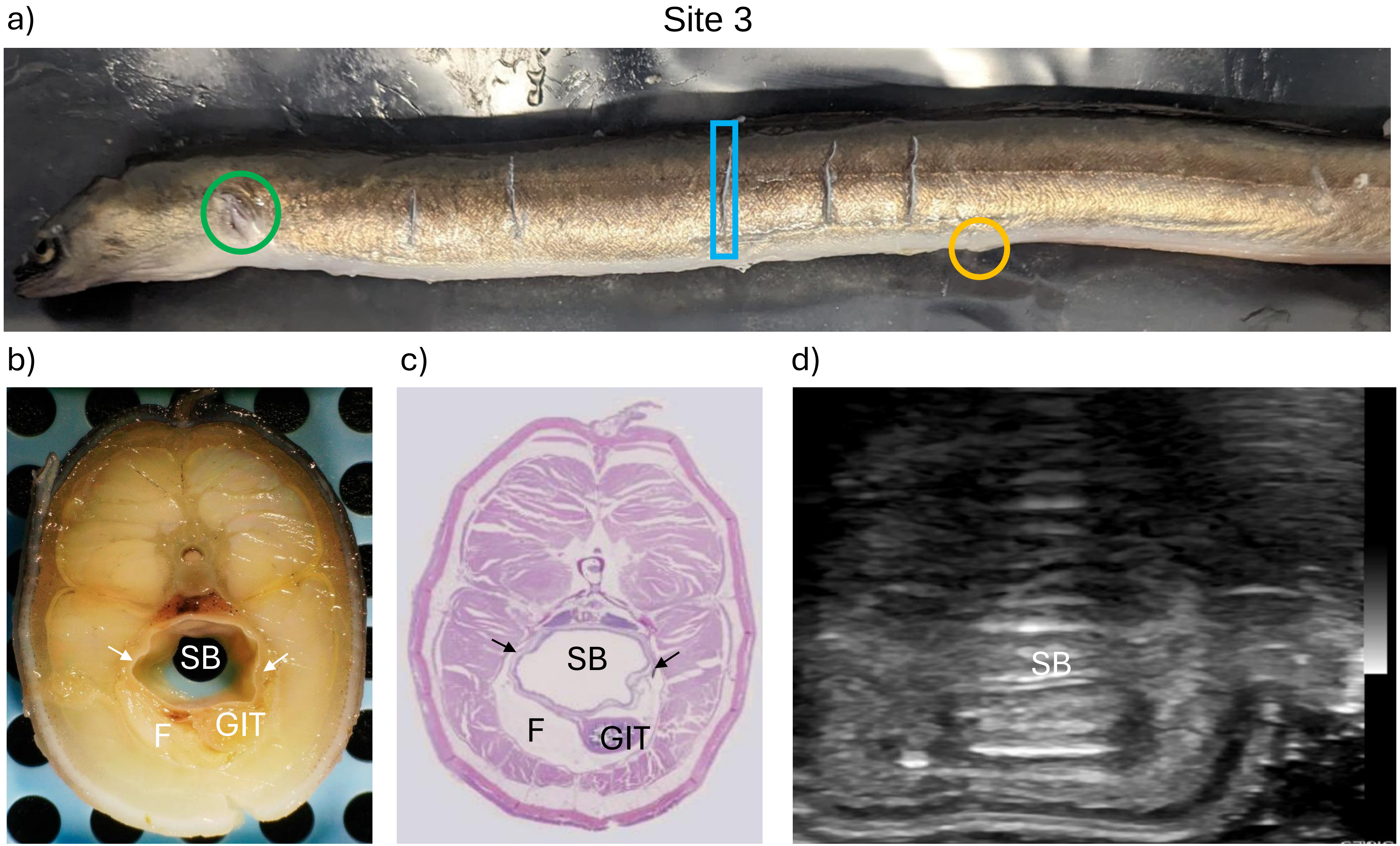

Figure S3. a) Image of male eel and location of scan at Site 3. Scanning site is highlighted in a blue rectangle, pectoral fin and gill vent highlighted in the green circle and anal pore located in yellow circle. Fixed section (b), histological section (c) and ultrasound image (d) at that site shown. Location of fat (F), gastrointestinal tract (GIT) and swim bladder (SB) indicated on all three images with presence of gonad indicated by white or black arrow. Small gonad size from fixed and histological sections and no gonad visible in ultrasound indicating that the individual is male.

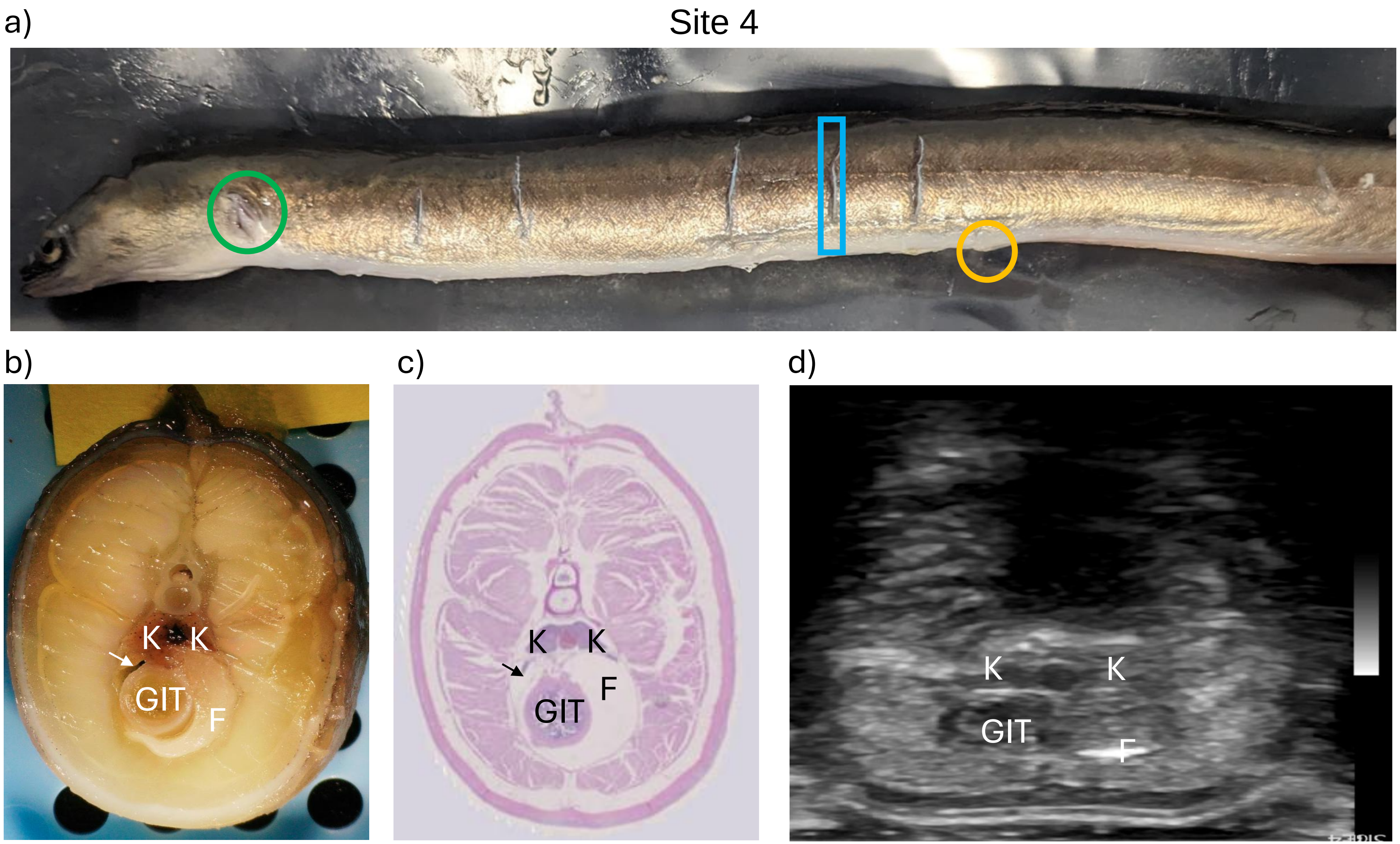

Figure S4. a) Image of male eel and location of scan at Site 1. Scanning site is highlighted in a blue rectangle, pectoral fin and gill vent highlighted in the green circle and anal pore located in yellow circle. Fixed section (b), histological section (c) and ultrasound image (d) at that site shown. Location of gastrointestinal tract (GIT), fat (F) and kidney (K) indicated on all three images with presence of gonad indicated by white or black arrow. Small gonad size from fixed and histological sections and no gonad visible in ultrasound indicating that the individual is male.

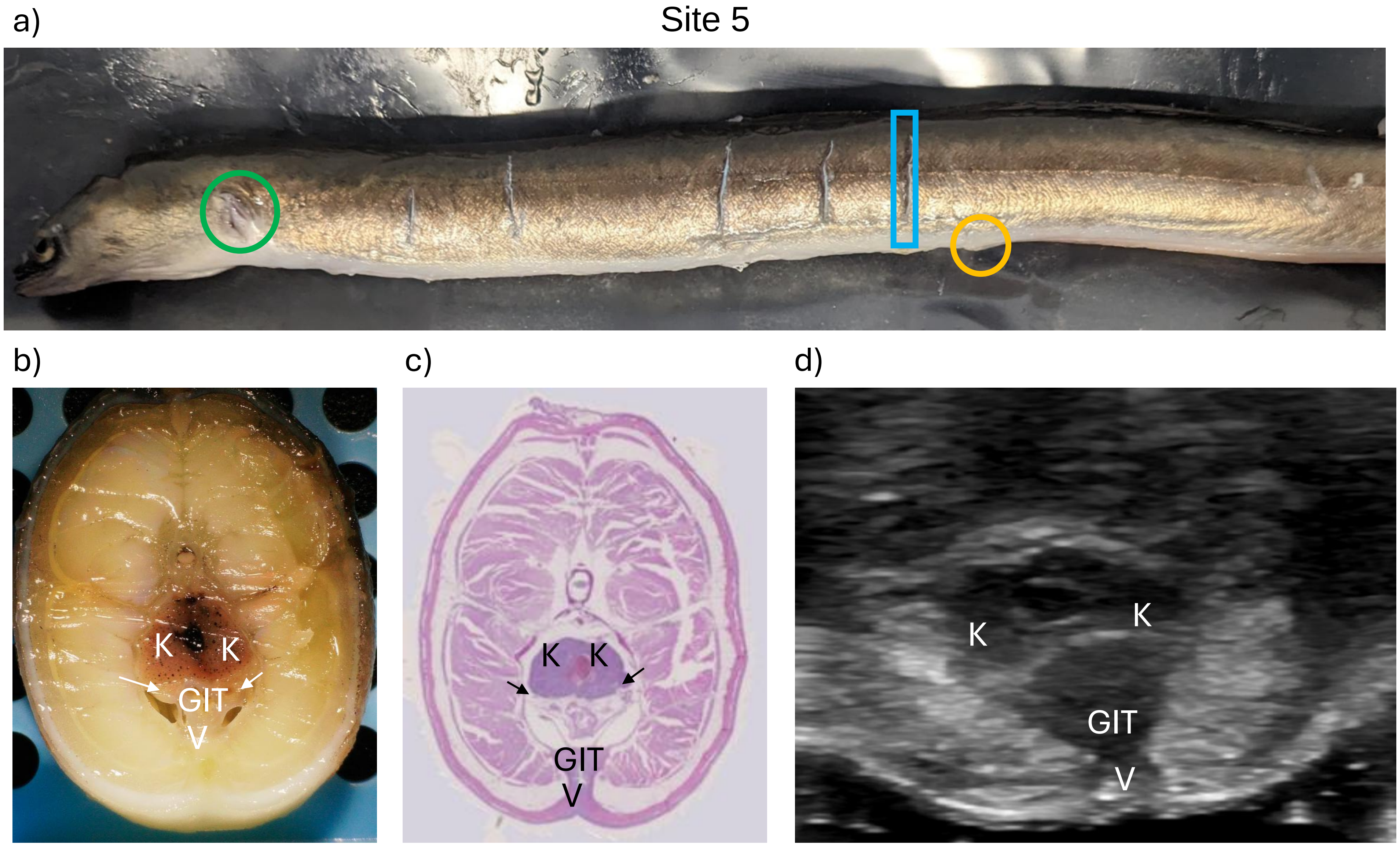

Figure S5. a) Image of male eel and location of scan at Site 1. Scanning site is highlighted in a blue rectangle, pectoral fin and gill vent highlighted in the green circle and anal pore located in yellow circle. Fixed section (b), histological section (c) and ultrasound image (d) at that site shown. Location of gastrointestinal tract (GIT), kidney (K) and anal pore/vent (V) indicated on all three images with presence of gonad indicated by white or black arrow. Small gonad size from fixed and histological sections and no gonad visible in ultrasound indicating that the individual is male.
